# A Two-Sample Mendelian Randomization Study of Neuroticism and Sleep Bruxism

**DOI:** 10.1177/00220345241264749

**Published:** 2024-08-26

**Authors:** T. Strausz, S. Strausz, S.E. Jones, T. Palotie, F. Lobbezoo, J. Ahlberg, H.M. Ollila

**Affiliations:** 1 Institute for Molecular Medicine Finland, Helsinki Institute of Life Science, University of Helsinki, Helsinki, Finland; 2 Department of Oral and Maxillofacial Diseases, Head and Neck Center, Helsinki University Hospital, Helsinki, Finland; 3 Cleft Palate and Craniofacial Center, Department of Plastic Surgery, Helsinki University Hospital and University of Helsinki, Helsinki, Finland; 4 Orthodontics, Department of Oral and Maxillofacial Diseases, Clinicum, Faculty of Medicine, University of Helsinki, Helsinki, Finland; 5 Department of Orofacial Pain and Dysfunction, Academic Centre for Dentistry Amsterdam (ACTA), University of Amsterdam and Vrije Universiteit Amsterdam, Amsterdam, The Netherlands; 6 Broad Institute of MIT and Harvard, Cambridge, MA, USA; 7 Center for Genomic Medicine, Massachusetts General Hospital, Boston, MA, USA; 8 Anesthesia, Critical Care, and Pain Medicine, Massachusetts General Hospital and Harvard Medical School, Boston, MA, USA

**Keywords:** Molecular Epidemiology, genome-wide association study, population register, anxiety, depression, psychologic stress

## Abstract

Sleep bruxism (SB) affects a considerable part of the population and is associated with neuroticism, stress, and anxiety in various studies. However, the causal mechanisms between neuroticism and SB have not been examined. Understanding the reasons for SB is important as understanding bruxism may allow improved comprehensive management of the disorders and comorbidities related to it. Previous studies on the association of risk factors to SB have provided important symptomatic insight but were mainly questionnaire based or limited in sample size and could not adequately assess causal relationships. The aim of this study was to elaborate the possible causal relationship of neuroticism as a risk factor for SB through a Mendelian randomization (MR) approach by combining questionnaires, registry data, and genetic information in large scale. We performed a two-sample MR study using instrumental genetic variants of neuroticism, including neuroticism subcategories, in the UK Biobank (*n* = 380,506) and outcome data of probable SB using FinnGen (*n* [cases/controls] = 12,297/364,980). We discovered a causal effect from neuroticism to SB (odds ratio [OR] = 1.38 [1.10–1.74], *P* = 0.0057). A phenotype sensitive to stress and adversity had the strongest effect (OR = 1.59 [1.17–2.15], *P* = 0.0028). Sensitivity analyses across MR methods supported a causal relationship, and we did not observe pleiotropy between neuroticism and SB (MR-Egger intercept, *P* = 0.87). Our findings are in line with earlier observational studies that connect stress and SB. Furthermore, our results provide evidence that neurotic traits increase the risk of probable SB.

## Introduction

Bruxism can occur during sleep (sleep bruxism [SB]) or during wakefulness (awake bruxism [AB]) (Lobbezoo et al. 2013). Even though the etiology of AB has remained unclear, SB may have several underlying factors, including stress sensitivity, anxiety, and depression (Manfredini and Lobbezoo 2009). Previous investigations connect SB to stress factors, and several studies have proposed SB to be potentially associated with psychosocial components, such as anxiety (Guo et al. 2018) and stress (Lavigne et al. 2003; Giraki et al. 2010). However, a review concluded that the current literature is controversial regarding an association between SB and generic symptoms of anxiety in adults (Polmann et al. 2019).

Neuroticism typically manifests as higher anxiety and stress. Neuroticism is often defined as a tendency for quick arousal and slow relaxation from arousal, particularly after a negative emotional trigger. Neuroticism can also be described as a poor ability to manage psychological stress. Overall, a low score in neuroticism reflects emotional stability and lower stress reactivity (DeNeve and Cooper 1998). Consequently, neuroticism is considered an important and informative phenotype in psychiatric genetic research because of its high genetic correlation with anxiety (r_g_ = 0.77) and depression (r_g_ = 0.60) (Hettema et al. 2006; Anttila et al. 2018) and as neuroticism can be assessed in individuals who do not meet the clinical diagnosis for either depression nor anxiety. Neuroticism is a heritable trait, with an estimated broad-sense heritability of 47% and no apparent signs of family environmental influences (Vukasović and Bratko 2015; Boomsma et al. 2018; Cheesman et al. 2020).

Neuroticism is often evaluated through questionnaires, either with the Eysenck Personality Questionnaire Revised Short Form (EPQ-RS; Eysenck et al. 1985) on 12 dichotomous (yes/no) neuroticism items or with the NEO Five-Factor Inventory (NEO-FFI; Costa and McCrae 2008) on 12 items with a scale of 1 to 5.

In a recent study, EPQ-RS neuroticism questionnaire items were shown to be genetically heterogeneous, with genetic overlap often being only moderate (i.e., r_g_ < 0.60). After a hierarchical clustering of genetic correlations of 33 external traits, two genetically homogenous item clusters in the EPQ-RS were identified (Nagel, Watanabe, et al. 2018). These clusters were denoted as depressed affect and worry, having especially strong genetic correlations with depression and anxiety, respectively (Nagel, Jansen, et al. 2018; Nagel, Watanabe, et al. 2018). In addition, a later study identified a third cluster containing three of the four remaining EPQ-RS neuroticism scale, called sensitivity to environmental stress and adversity (SESA; Nagel et al. 2020).

Most causality analyses in epidemiology are based on randomization (Moher et al. 2010). However, many exposures cannot be randomly allocated or repeated for practical or ethical reasons.

As humans have two pairs of each chromosome, each individual carries two genetic variants for each position of the genome. However, only one of the genetic variants is passed to children, and these genetic variants are inherited randomly. Sophisticated models such as Mendelian randomization (MR) leverage randomly inherited alleles and use them to assess causality, which cannot be estimated through traditional observational epidemiological studies (Smith and Ebrahim 2003; Davey Smith and Hemani 2014). Therefore, in the MR setting, the single nucleotide polymorphisms (SNPs) used for the two-sample MR method can be used to identify variant-exposure associations from one genome-wide association study (GWAS) and variant-outcome associations from another GWAS. The method leverages publicly available summary data, which provide sufficient statistical power for robust associations. Consequently, small causal effects of common genetic variants termed *exposure variables* can be used to infer causality (Burgess et al. 2015). In this study, we examined the causal relationship of neuroticism as a risk factor for SB through two-sample MR (Corbin et al. 2016; Haycock et al. 2016).

## Materials and Methods

### Study Design

#### Two-sample MR analysis

We used summary data from previously published GWAS on the neuroticism EPQ-RS questionnaire and its subcategories from the UK Biobank (UKBB; Nagel, Jansen, et al. 2018; Nagel, Watanabe, et al. 2018; Nagel et al. 2020) publicly available through the IEU GWAS database (https://gwas.mrcieu.ac.uk) for the exposure instrumental variable selection. For all exposures, we selected all reported independent lead variants (with association *P* < 5 × 10^−8^) in the discovery GWAS as instruments and used the same study for both instrument selection and effect size determination. We used these instruments to the GWAS of probable SB, which we had generated earlier in the FinnGen project (Strausz et al. 2023).

**Figure 1.**
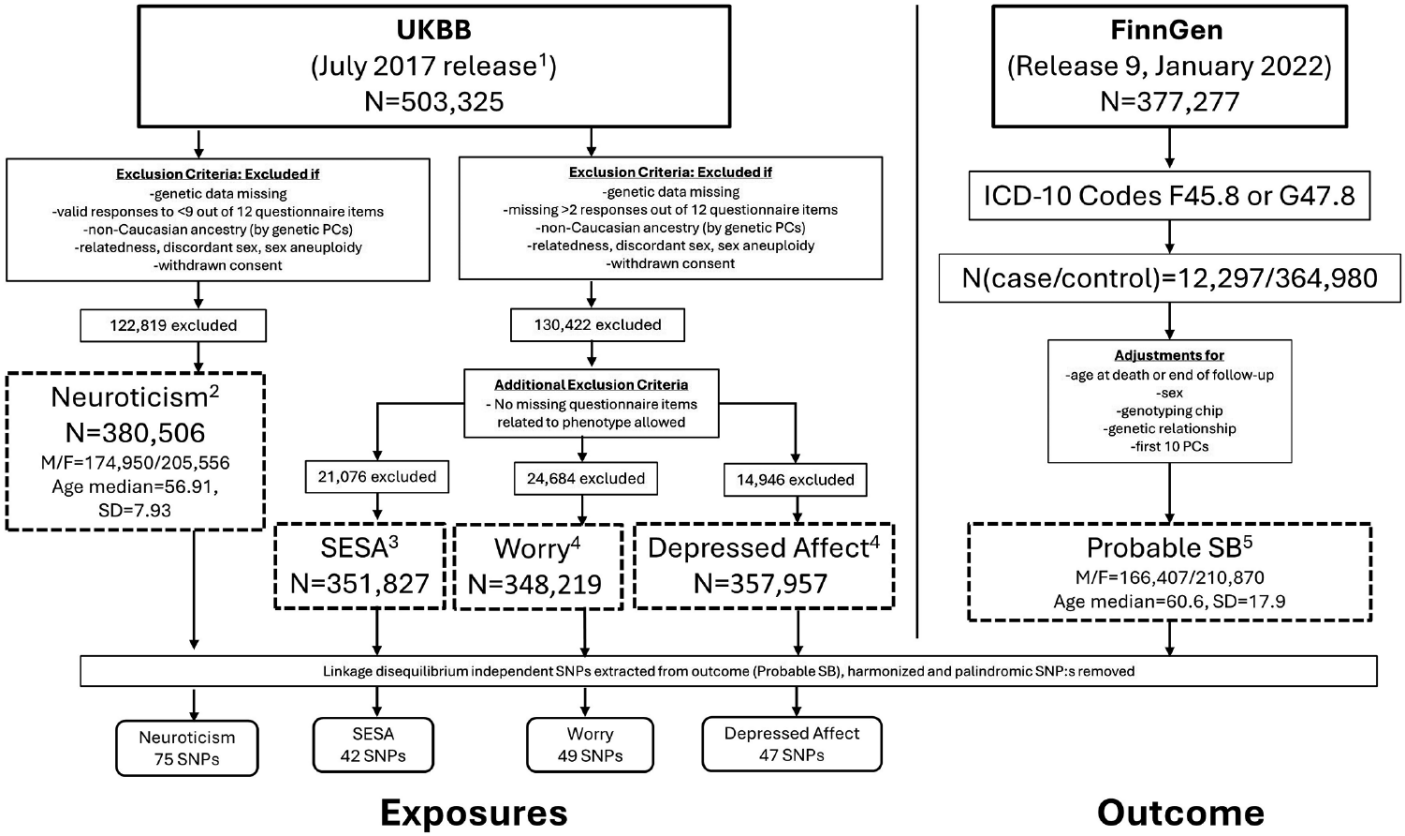
Flow chart representation of the formation of cohorts used in the two-sample Mendelian randomization analyses. Dashed boxes represent the genome-wide association studies, with superscript numbers pointing to the respective publications. F, female; ICD, International Classification of Diseases; M, male; PC, principal component; SB, sleep bruxism; SD, standard deviation; UKBB, UK Biobank. 1. Bycroft et al. 2018. 2. Nagel, Watanabe, et al. 2018. 3. Nagel et al. 2020. 4. Nagel, Jansen, et al. 2018. 5. Strausz et al. 2023.

As the exposure variables are based on the UKBB cohort and the outcome variable summary statistics derive from FinnGen with only Finnish-ancestry samples, there is no overlap between exposure and outcome cohorts. A brief visualization on the formation of cohorts and GWAS analyses is represented in Figure 1. Additional information on the FinnGen and UKBB cohorts, including data access and the assumptions of MR studies can be found in the Appendix (Pages 2-5, Supplementary Tables 1-2).

TwoSampleMR (https://github.com/MRCIEU/TwoSampleMR) version 0.5.6 was used to generate a list of variants that represented independent signals (in other words, linkage disequilibrium–independent instrumental variables) for each exposure and extracted them from probable SB risk (outcome). Exposure and outcome data were harmonized and palindromic SNPs removed, resulting in 75 genetic instruments for neuroticism, 49 for worry, 47 for depressed affect, and 42 for SESA (Supplementary Tables 3–6).

The SNP-exposure and SNP-outcome associations were assessed using three methods with different assumptions: inverse-variance weighted approach (IVW; Lawlor et al. 2008), weighted median approach (Bowden et al. 2016), and MR-Egger regression (Bowden et al. 2015). We also estimated MR-Egger intercept to test pleiotropy and performed leave-one-out analysis to estimate the robustness of the causal association. The main MR analyses and sensitivity analyses were run using TwoSampleMR. We performed statistical analyses using R version 4.2.1. Individuals with missing data were excluded from GWAS analyses. Variants without available association statistics in either the exposure or outcome were not included in the analysis for that exposure-outcome pair. Multiple testing was adjusted by Bonferroni correction if several statistical tests were performed simultaneously on a single data set. We considered there to be evidence of a causal association if the IVW estimate was significant at a Bonferroni-adjusted threshold of *P* < 0.05 and if the less well-powered, but pleiotropy-robust, weighted median and MR-Egger estimates were directionally consistent with the IVW estimate. A statistically significant MR-Egger intercept term (*P* < 0.05) was considered as evidence of directional pleiotropy. Quantitative variables formed by summing binary questionnaire items (neuroticism, worry, depressed affect, SESA) were z-score normalized before running the GWAS analysis, and a linear regression model with a simple additive model was used.

### Study Cohorts

#### Outcome

##### Probable SB

To identify individuals in the FinnGen cohort with probable SB, International Classification of Diseases, 10th revision (ICD-10) codes F45.8 “Other Somatoform Disorders” (*n* = 11,957) and G47.8 “Other Sleep Disorders” (*n* = 289) were extracted from the Finnish national hospital and primary care registries. A diagnosis based on a positive self-report and positive clinical findings should be graded as “probable” SB in research settings (Lobbezoo et al. 2013). As the signs of SB may be quite reliably detected and reported by dentists (Abe et al. 2009) and a diagnosis requires a positive self-report in addition to clinical signs, our phenotype represents probable SB (Strausz et al. 2023).

A GWAS analysis of 377,277 individuals from the FinnGen release R9 (released publicly in April 2022) linked with Finnish hospital and primary care registries identified 12,297 (3.26%) probable SB cases (*n*(F45.8) = 11,957, *n*(G47.8) = 289, *n*(F45.8&G47.8) = 51) through extracting these ICD-10 codes for SB from electronic health records. Cases were those participants with at least one of the above (case inclusion) diagnosis codes, and controls were those who were not cases. These diagnoses were used to create the binary outcome “probable SB” (Strausz et al. 2023). After quality controlling, a total of 20,861,210 imputed SNPs were available. The GWAS analysis was performed using REGENIE23 v2.2.4 and adjusted for sex, genotyping chip, genetic relationship, the first 10 genetic principal components, and age at follow-up end (October 11, 2021) or death, as the FinnGen endpoint diagnosis data extend beyond the initial recruitment visit (Fig. 1).

#### Exposures

##### Neuroticism phenotype and subcluster classifications

**Figure 2.**
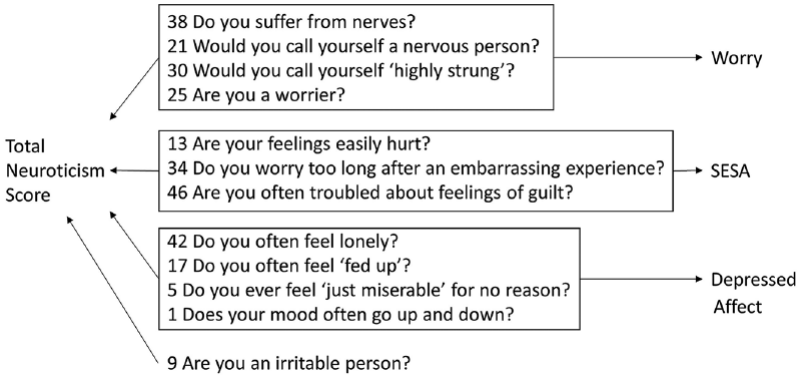
Representation of the Eysenck Personality Questionnaire Revised Short Form (EPQ-RS) questionnaire items (item numbers are depicted before the questions) used for neuroticism and subcluster phenotypes. SESA, sensitivity to stress and adversity.

In all UKBB cohorts, neuroticism was assessed by the EPQ-RS (Eysenck et al. 1985) neuroticism scale questions (Fig. 2), in which respondents answered yes or no (score 1 or 0, respectively) to each question to sum the total neuroticism score for each respondent between 0 and 12. This short scale has a reliability of more than 0.8 for neuroticism and high concurrent validity (correlation *r* = 0.85) when compared with the internationally most widely used personality assessment scale NEO Five-Factor Inventory (NEO-FFI; Gow et al. 2005).

GWAS analyses of the UKBB neuroticism phenotypes were implemented in PLINK 1.9 using linear regression to analyze the neuroticism sum score and item cluster scores. Sex, age, genotype array, and Townsend deprivation index (a measure based on postal code indicating material deprivation) were included as covariates. In addition, the first 10 genetic principal components (PCs) were included as covariates to control for potential population stratification. Genetic PCs were computed using FlashPCA 2 on individuals of European ancestry, after linkage disequilibrium pruning and filtering out SNPs with a minor allele frequency (MAF) of <0.01 and genotype missingness of >0.05. Final data analysis was restricted to autosomal, biallelic SNPs with MAF > 0.0001, high imputation quality (INFO score ≥ 0.9), and low missingness (<0.05), resulting in 10,847,151 SNPs. The standard genome-wide significance threshold in European-descent samples of *P* < 5 × 10^−8^ was applied within each SNP-based GWAS analysis.

##### Neuroticism

For the neuroticism phenotype, UKBB data (Nagel, Watanabe, et al. 2018) were used, in which neuroticism was measured with 12 dichotomous (yes or no) items of the EPQ-RS. Responders with fewer than 9 answered items, who did not have valid genetic data or were not of European descent, were excluded from analyses, resulting in a sample size of 380,506 individuals with both phenotypic and genetic data. A weighted sum score was calculated by adding individual valid item responses (mean = 4.16, SD = 3.21) and dividing that sum by the total number of valid responses, ranging from 0 to 12.

##### Worry

The previously defined phenotype of the neuroticism subcluster of the EPQ-RS “worry” included only participants (*n* = 348,219) who answered to all following questions: “Are you a worrier?”, “Do you suffer from nerves?”, “Would you call yourself a nervous person?”, and “Would you call yourself tense or highly strung?”, and the scores (0 or 1) on these four EPQ-RS items were summed (with a result between 0 and 4; Nagel, Jansen, et al. 2018). The mean score for the worry cluster was 1.16 (SD = 1.25) and was higher in females compared with males (Cohen’s *d* = 0.22).

##### Depressed affect

Questions “Do you often feel lonely?”, “Do you ever feel ‘just miserable’ for no reason?”, “Does your mood often go up and down?”, and “Do you often feel ‘fed up’?” were used to obtain scores for the EPQ-RS “depressed affect” cluster, and the sum of scores (between 0 and 4) was used for analysis (*n* = 357,957; Nagel, Jansen, et al. 2018). The mean score for the depressed affect cluster was 1.45 (SD = 1.42) and was higher in females compared with males (Cohen’s *d* = 0.20).

##### SESA

Three of the remaining four questions with a common theme, namely, “Are your feelings easily hurt?”, “Do you worry too long after an embarrassing experience?”, and “Are you often troubled by feelings of guilt?”, formed the EPQ-RS “SESA” cluster, and the sum of scores (between 0 and 3) was used for analysis (*n* = 351,827; Nagel et al. 2020).

## Results

### Neuroticism Increases the Risk for SB

**Figure 3.**
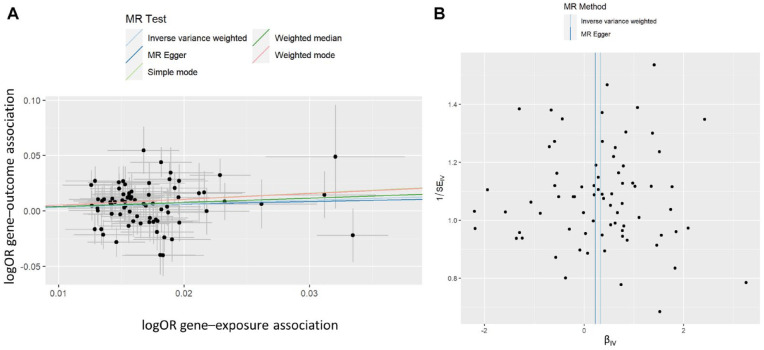
Genetic associations of probable sleep bruxism (SB) with neuroticism score. Analysis of 380,506 individuals with 12 dichotomous (yes or no) items of the Eysenck Personality Questionnaire Revised Short Form using 75 single nucleotide polymorphisms (SNPs) after harmonization of a possible 91 SNPs. (**A**) Scatter plot showing the correlation of genetic associations of the neuroticism score (*x*-axis) with genetic associations of probable SB (*y*-axis). Lines represent the slopes of the different regression analyses. (**B**) Funnel plot showing Mendelian randomization (MR) causal estimate (*x*-axis) and the inverse variance (*y*-axis) correlations, that is, causal estimates against their precision, for each SNP in the MR analysis. Lines represent the effect sizes of the different regression analyses. Estimates correspond to an odds ratio (OR = e^β^) for probable SB per unit increase in neuroticism score of 1.38 (95% confidence interval [CI] 1.10–1.74), 1.47 (95% CI 1.08–2.01), and 1.25 (95% CI 0.36–4.36) for the inverse-variance weighted IVW, weighted median, and MR-Egger analyses, respectively.

After harmonization, 75 genetic instruments that were previously identified in the UK Biobank and had robust independent significant associations in the original study (*P* < 5 × 10^−8^, Supplementary Tables 3–6) were used for the analysis. Two-sample MR analysis, using neuroticism as an exposure variable (predisposing) and probable SB as an outcome, indicated a causal relationship, with neuroticism increasing the risk for SB (odds ratio [OR] 1.38, 95% confidence interval (CI) 1.10–1.74, *P* = 0.0057, Fig. 3).

**Figure 4.**
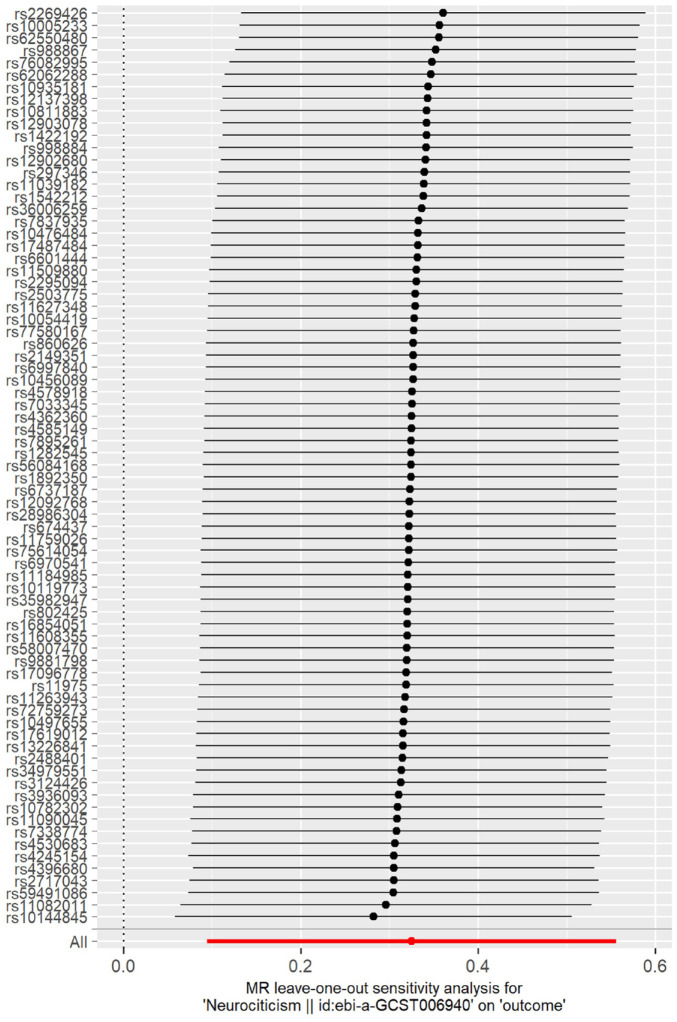
Mendelian randomization leave-one-out analysis for neuroticism on probable sleep bruxism. Each black line in the figure corresponds to the outcome of the MR analysis when one single nucleotide polymorphism (SNP; indicated on the left) is removed from the analysis, while the remaining SNPs are used. The red line indicates the overall effect. There is no evidence for outliers weighing on the total causal estimate.

Two alternative MR methods provided evidence of a causal relationship between neuroticism score and probable SB, where the estimate’s *P*-value was significant (Table), and effect sizes were consistently positive across all tested methods. This is demonstrated in Figure 3A, where the slope of the lines shows the causal effect estimates as predicted by the IVW, MR-Egger, and weighted median approaches, although the MR-Egger approach did not reach statistical significance. Furthermore, to understand the robustness of the signal and the variants, we estimated how individual variants contributed to the signal from the MR analysis (Fig. 4, Supplementary Figure 1). We did not observe any outliers in the distribution of effects shown in Figures 3B and 4. Similarly, there was no evidence of directional horizontal pleiotropy as indicated by the MR-Egger intercept of 1.73 × 10^−3^ (*P* = 0.87) in the Egger analysis. Finally, we examined the different components of neuroticism and observed that SESA and worry causally associated with probable SB (Table), suggesting an association with more than one component of neuroticism with SB. The results for neuroticism subclusters are further illustrated by scatterplots and leave-one analyses in the Appendix (Supplementary Figures 2-4).

**Table.** Mendelian Randomization Results.

Exposure (Item Count)	Neuroticism (12)	Worry (4)	Depressed Affect (4)	SESA (3)
SNP count (*P* < 5 × 10^−8^, harmonized)	75	49	47	42
Sample size	380,506	348,219	357,957	351,827
IVW, OR [95% CI]	1.38 [1.10–1.74]	1.38 [1.01–1.88]	1.36 [0.97–1.92]	1.59 [1.17–2.15]
*P* value	**0.0057**	**0.040**	0.076	**0.0028**
Weighted median, OR [95% CI]	1.47 [1.08–2.01]	1.42 [0.99–2.04]	1.28 [0.85–1.92]	1.74 [1.17–2.58]
*P* value	**0.016**	0.055	0.24	**0.0063**
MR Egger, OR [95% CI]	1.25 [0.36–4.36]	1.92 [0.41–8.96]	−0.53 [0.13–2.72]	1.32 [0.28–6.25]
*P* value	0.73	0.41	0.50	0.73
Egger intercept [SE]	0.0017 [0.011]	−0.0060 [0.014]	0.014 [0.0126]	0.0031 [0.01295]
*P* value	0.87	0.67	0.28	0.81

Estimates from the application of inverse-variance weighted, weighted median, and MR-Egger Mendelian randomization methodologies. Values represent the estimated causal effect of exposure phenotypes on probable sleep bruxism (SB). Egger intercept coefficients represent the average pleiotropic effect of a genetic variant on probable SB risk, with nonsignificant *P* values (*P* > 0.05) indicating no signs of pleiotropy. CI, confidence interval; IVW, inverse variance weighted; OR, odds ratio; SESA, sensitivity to stress and adversity; SNP, single nucleotide polymorphism. Bold indicates *P* < 0.05.

## Discussion

In this article, we examined if neuroticism is related to SB. We showed a causal relationship from neuroticism to probable SB supporting earlier observational findings that connect neuroticism, anxiety, and depression with SB in observational studies. Our results show a statistically significant result between neuroticism and probable SB with a robust causal estimate across two MR methods. These findings indicate that in addition to comorbid traits, neuroticism and SB are likely causally related so that neuroticism increases the risk for SB as our results show that immutable common genetic variants strongly associated with increasing neuroticism scores seem to also increase the probability for SB in a genetically separate cohort. Our findings are in line with insights from clinical practice as well as earlier epidemiological studies and bring a novel causal estimate into the field of bruxism research.

Previous epidemiological studies suggest SB has associations with sleep disorders (Kuang et al. 2022), behavioral traits, and pharmacologic treatments, in addition to psychological traits (Ahlberg et al. 2020). However, the assessment of associations between complex traits such as anxiety (Polmann et al. 2019) and SB benefits from larger samples and causality estimates to better provide information into the connections between SB and psychological factors.

Observational studies alone cannot determine the causality of even strong epidemiological associations because it is often impossible to rule out residual confounding or reverse causation. Even when robust associations have been tested experimentally, they may prove to have been merely spurious findings (Smith and Ebrahim 2003). MR methods have been previously used to study the question of causality in complex traits, such as coronary heart disease (Holmes et al. 2015; Richardson et al. 2020), where cross-sectional and longitudinal studies are often troubled by these sources of variation (Smith and Ebrahim 2003; Davey Smith and Hemani 2014). SB is a complex trait, in which causal risk factors have largely remained unidentified.

Neuroticism is a heritable personality trait, characterized by negative emotions such as worrying, feelings of guilt, loneliness, and being easily hurt. Increased levels of neuroticism are associated with poor mental health (Jeronimus et al. 2016). Several earlier studies have examined the relationship between neuroticism and SB. For example, a 2009 review concluded that AB, but not SB, seems to be related to psychological factors. However, most of the assessed articles using clinical dental examination (indicating probable SB) had concluded that psychological factors did associate with bruxism (Manfredini and Lobbezoo 2009). During these earlier studies, the consensus on differentiating between AB and SB in research was still emerging, and many studies were heterogenous in their definitions. In contrast, more recent studies using current bruxism definitions and methods more resistant to confounding have consistently found associations of self-reported SB and anxiety (Chattrattrai et al. 2022). In addition, other metrics such as cortisol levels (Fritzen et al. 2022) and autonomic nervous system responses in polysomnographic studies (Shiraishi et al. 2021) support the association of SB and stress and point to the biological mechanisms linking neuroticism to SB. The diagnostic criteria, assessment methods, and metrics regarding bruxism continue to evolve, and once established, they will most likely reflect changes in the way previous associations are viewed and emphasize the need for further studies (Manfredini et al. 2019; Lavigne et al. 2021; Manfredini et al. 2024).

Both the earlier and more recent studies found a connection between bruxism and psychological factors, providing a broad scientific basis for the overall connection between bruxism and psychological traits. Our study builds on top of this earlier literature connecting neuroticism and SB in a causal fashion.

### Strengths and Limitations

In this study, large-scale data and modern genetic methods were used to strengthen the previously held view of a causal relationship between anxiety and stress with SB.

Neuroticism, as a personality trait, is useful in evaluating an individual’s sensitivity to stress and susceptibility to anxiety and depression, as otherwise stressors in life vary across time. Dissecting neuroticism into genetically homogenous clusters adds robustness to our results, because if questionnaire items were heterogeneous, statistical power may be lost when converted into a continuous variable.

The effect size of the exposure to outcome is not typically the focus of standard MR studies, as genetic instruments usually explain only a very small proportion of the variance in the exposure of interest. This stands true also in our study, and to reflect the effect of one additional positive questionnaire in increasing the odds of a probable SB diagnosis in a hypothetical model was therefore not explored in our study. MR estimates the causal effect assuming the IV assumptions hold. It is possible that, due to the large sample size of the UKBB neuroticism assessment, modifying it into a quantitative variable, and thus the high power to detect genetic associations, a proportion of the selected instruments may be secondary associations for neuroticism, being associated with a phenotype that itself influences neuroticism scores. MR-Egger regression can help evaluate how much pleiotropy might bias the estimates of variance explained, and in our study’s case, there was no evidence of pleiotropy.

We used the only currently available GWAS of probable SB as the outcome (Strausz et al. 2023). This GWAS is based on two ICD-10 diagnoses from electronic health records without instrumental evaluation. As the registry data in Finland also provides information on the medical specialty of the doctor that has assigned the diagnosis, although having been mainly coded by dentists, in some circumstances these diagnosis codes might also relate to other rare pathologies outside SB, because ICD-10 diagnoses F45.8 and G47.8 are not specific for the condition. Considering the diagnostic grading of bruxism (Lobbezoo et al. 2013), possible SB, probable SB, and definite SB may be thought of as genetically different phenotypes altogether, and therefore our results may not translate directly as an interpretation of causality with definite, or possible, SB.

We were unable to explore potential reverse causation using a bidirectional MR design, because of only one genome-wide scale statistically significant SNP (*P* < 5 × 10^−8^) in the probable SB GWAS.

The GWAS analyses used in the MR were performed entirely in (FinnGen, UKBB) individuals with European ancestry. The lack of diverse ancestral representation in large biomedical cohorts and publicly available GWAS data remains one of the greatest limitations of the field, and therefore, we cannot comment on the applicability of our findings to other ancestries. However, the findings should be generalizable across other exposure levels and timings.

## Conclusion

MR methods show a causal relationship from neuroticism questionnaire scores to a probable SB diagnosis across sensitivity analyses. After separately evaluating the genetically more homogenous questionnaire item clusters, we found that especially a personality type sensitive to adversity and stress is more causally associated with having a probable SB diagnosis. Our results support the previous evidence for stress and anxiety as a causal risk factor for SB and underscore the importance of psychological screening for comprehensive care. As the results imply a directional causality from increased stress sensitivity to clinically significant SB, techniques and lifestyle alterations to reduce psychological stress might be useful for alleviating negative outcomes resulting from SB, and further randomized controlled trials with psychological interventions such as cognitive-behavioral therapy are suggested.

## Author Contributions

T. Strausz, contributed to conception and design, data acquisition, analysis, and interpretation, drafted and critically revised the manuscript; S. Strausz, contributed to conception and design, data acquisition and analysis, critically revised the manuscript, S.E. Jones, contributed to data analysis and interpretation, critically revised the manuscript; T. Palotie, contributed to design, critically revised the manuscript; F. Lobbezoo, J. Ahlberg, contributed to design, data interpretation, critically revised the manuscript; H.M. Ollila, contributed to conception and design, data analysis and interpretation, drafted and critically revised the manuscript. All authors gave their final approval and agree to be accountable for all aspects of the work.

## Supplemental Material

sj-docx-1-jdr-10.1177_00220345241264749 – Supplemental material for A Two-Sample Mendelian Randomization Study of Neuroticism and Sleep Bruxism Supplemental material, sj-docx-1-jdr-10.1177_00220345241264749 for A Two-Sample Mendelian Randomization Study of Neuroticism and Sleep Bruxism by T. Strausz, S. Strausz, S.E. Jones, T. Palotie, F. Lobbezoo, J. Ahlberg and H.M. Ollila in Journal of Dental Research
