# Supplementary material for: A Two-Sample Mendelian Randomization Study of Neuroticism and Sleep Bruxism
Source: J Dent Res. 2024 Aug 26;103(10):980–7. doi: 10.1177/00220345241264749 (PMC11409563; doi:10.1177/00220345241264749)
Supplement: sj-docx-1-jdr-10.1177_00220345241264749 – Supplemental material for A Two-Sample Mendelian Randomization Study of Neuroticism and Sleep Bruxism [file sj-docx-1-jdr-10.1177_00220345241264749.docx]

*Supplementary material*

**A Two-sample Mendelian Randomization Study of Neuroticism and Sleep Bruxism**

Tommi Strausz^1^, Satu Strausz^1,2,3^, Samuel E. Jones^1^, Tuula Palotie^2,4^, Frank Lobbezoo^5^, Jari Ahlberg^2^, Hanna M. Ollila^1,6,7,8^

1. Institute for Molecular Medicine Finland, Helsinki Institute of Life Science, University of Helsinki, Helsinki, Finland
2. Department of Oral and Maxillofacial Diseases, Head and Neck Center, Helsinki University Hospital, Helsinki, Finland
3. Cleft Palate and Craniofacial Center, Department of Plastic Surgery, Helsinki University Hospital and University of Helsinki, Helsinki, Finland
4. Orthodontics, Department of Oral and Maxillofacial Diseases, Clinicum, Faculty of Medicine, University of Helsinki, Helsinki, Finland
5. Department of Orofacial Pain and Dysfunction, Academic Centre for Dentistry Amsterdam (ACTA), University of Amsterdam and Vrije Universiteit Amsterdam, Amsterdam, The Netherlands
6. Broad Institute of MIT and Harvard, Cambridge, Massachusetts, USA
7. Center for Genomic Medicine, Massachusetts General Hospital, Boston, USA
8. Anesthesia, Critical Care, and Pain Medicine, Massachusetts General Hospital and Harvard Medical School, Boston, USA

Corresponding author: Hanna M. Ollila

PL 20 (Tukholmankatu 8), 00014, Finland

[hanna.m.ollila@helsinki.fi](mailto:hanna.m.ollila@helsinki.fi)

**Additional descriptions of cohorts**

**FinnGen**

FinnGen (www.finngen.fi/en) is a research project of Finnish residents, forming a population-based cohort from newborn to 104 years old at baseline recruitment, that have consented to participate in Finnish regional biobanks. The project combines genetic data with electronic health record data derived from nationwide primary care registers, hospital in- and out-patient visits and prescription information. The aim is to understand the genetic etiology of, and drive the development of drugs to treat, a wide variety of diseases and disorders. FinnGen release 9 (R9) contains health and genetic data on up to 392,396 participants primarily of Finnish ancestry. Diagnosis data extraction from the public healthcare records is ongoing with data being released regularly. When a study participant has been recruited, their entire medical record is linked into the FinnGen database allowing comprehensive assessment of their medical history. Data collection for samples started in 2017 with 50,000 individual increases every six months. The project goal of 500,000 individuals was reached in 2023. Once samples were part of FinnGen, the genotype data was merged with electronic health record data, where national registries with diagnostic data started in 1969. All participants continue to be followed up unless they die (follow-up ends at death), or they withdraw their consent from the study (their data is removed entirely and is not used in analyses). As a large part of individuals have been invited to partake during treatment center or hospital visits, the study population has been noted to be enriched in older individuals, diseased phenotypes and involves more women than men compared to a random population sample.

All FinnGen participants provided informed consent for biobank research based on the Finnish Biobank Act (FBA). Prior to the FBA coming into effect (September 2013), participants recruited into the individual research cohorts provided study-specific consent for research. These consent permissions were transferred to the Finnish biobanks, at the conception of FinnGen in August 2017, after approval by Fimea (the Finnish Medicines Agency), the National Supervisory Authority for Welfare and Health. The recruitment protocols followed the biobank protocols approved by Fimea. The Coordinating Ethics Committee of the Hospital District of Helsinki and Uusimaa (HUS) statement number for the FinnGen study is Nr HUS/990/2017.

**UKBB**

The UK Biobank (UKBB) is a large-scale biomedical prospective database and research resource incorporating over 500,000 mainland UK population participants, aged between 37 and 73 at recruitment. Since 2006, UKBB has regularly collected biological and medical data as well as detailed information about lifestyle and personal charecteristics from individuals with ther consent. Participants were invited to undertake a baseline interview between 2006 and 2010 where data was collected on a variety of health and lifestyle measures, and where blood and urine samples were also taken for genetic and biochemistry analysis. Electronic health records, consisting of Hospital Episode Statistics inpatient (HES; max. N=440,512) and primary care (GP; max. N=231,364) were later linked up to provide longitudinal data on disease diagnosis, operations, medications, and deaths. Recent diagnoses are frequently added to the records and the participants’ entire medical history is made available, and they continue to be followed-up unless they die or withdraw their consent. While the UK Biobank participants are drawn from the general UK population, the study has some recruitment bias and as such participants are on average healthier, more educated and less deprived than the average UK resident.

The UK Biobank has received approval as a Research Tissue Bank from the North West Multi-centre Research Ethics Committee (MREC) under MREC permits 11/NW/0382 (2011-2016), 16/NW/0274 (2016-2021) and 21/NW/0157 (2021-2026). Researchers with approved applications are covered by these permits and are not required to seek additional approval, except in specific cases (see section B7 of the UK Biobank Access Procedures document: https://www.ukbiobank.ac.uk/media/omtl1ie4/access-procedures-2011-1.pdf). All participants of the UK Biobank study provided consent, at the baseline visit, for their personal data and biological samples to be collected and stored for research purposes. Participants are given the option to withdraw their consent at any time; any samples that have withdrawn their consent at the time of analysis were excluded from this study. A print version of the electronic consent form is stored as UK Biobank Resource 100252.

**Data access**

Individual-level data can be accessed on successful application to FinnGen and the UK Biobank cohorts. For FinnGen, applications for individual-level data can be made through the Finnish Biobanks’ “FinBB” portal (https://finbb.fi/) and summary GWA data, including the probable SB phenotype, can be accessed through the FinnGen website (https://www.finngen.fi/en/access_results). For the UK Biobank, applications for individual-level data can be made through the UK Biobank portal at https://www.ukbiobank.ac.uk/enable-your-research/apply-for-access. The FinnGen R9 GWA summary statistics for probable SB will become available to researchers in Q4 2024 at https://r9.finngen.fi/.

**MR assumptions**

For MR studies to validly show causality, three key assumptions - called the instrumental variable (IV) assumptions - must be met: 1) Relevance: The genetic variants (IVs) must be robustly associated with the exposure of interest. This ensures that the IVs can actually influence the exposure. 2) Independence: The genetic variants must be independent of confounders that affect both the exposure and the outcome. 3) Exclusion restriction: The genetic variants affect the outcome only through their effect on the exposure, not via any other pathway (no pleiotropy).

**Supplementary Tables**

**Supplementary Table 1.** Descriptive demographics of probable sleep bruxism GWAS cohort in FinnGen R9.

|  | All, N=377277 | Bruxism  N=12297 | No bruxism  N=364980 | OR [95%] | P |
| --- | --- | --- | --- | --- | --- |
| Sex |  |  |  |  |  |
| Male | 166407(44.1%) | 2476(20.1%) | 163931(44.9%) | 1 |  |
| Female | 210870(55.9%) | 9821(79.9%) | 201049(55.1%) | 2.78 [2.66-2.91] | <1x10^-300^ |
| Age in years (SD) | 60.6 (17.9) | 51.0 (15.8) | 60.9 (17.8) | 0.97 [0.97-0.98] | <1x10^-300^ |

Odds ratios (ORs) and P-values have been adjusted for age at death or end of follow-up, sex and first 10 principal components. OR = Odds ratio, SD = Standard deviation

**Supplementary Table 2.** Summary statistics for phenotypic variables Depressed affect and Worry of the UK Biobank sample of N=372,903.

| Phenotype | Nmissing | Total | Males | Females |
| --- | --- | --- | --- | --- |
|  |  | Mean (SD) | | |
| Depressed affect | 14,946 | 1.45  (1.42) | 1.29  (1.38) | 1.58  (1.45) |
| Worry | 24,684 | 1.16  (1.25) | 1.01  (1.22) | 1.28  (1.27) |

SD = Standard deviation

| **Supplementary Table 3.** Genetic instrumental variables used for two-sample MR analyses for Neuroticism and probable sleep bruxism. EA=effect allele, OA=other allele | | | | | | | | |
| --- | --- | --- | --- | --- | --- | --- | --- | --- |
|  |  |  |  |  |  |  |  |  |
|  |  |  | **Exposure (Neuroticism)** | | | **Outcome (Probable Sleep Bruxism)** | | |
| **Variant** | **EA** | **OA** | **Beta** | **SE** | **P-value** | **Beta** | **SE** | **P-value** |
| rs10005233 | T | C | 0.0146127 | 0.0023023 | 2.18998e-10 | -0.0283521 | 0.013227 | 0.032073 |
| rs10054419 | G | T | -0.0131218 | 0.00231302 | 1.40001e-08 | -0.000384084 | 0.013755 | 0.977723 |
| rs10119773 | G | A | 0.0160955 | 0.00235555 | 8.33489e-12 | 0.0095673 | 0.0131999 | 0.468574 |
| rs10144845 | T | C | 0.0181806 | 0.00245849 | 1.41286e-13 | 0.0440137 | 0.0134845 | 0.00109842 |
| rs10456089 | A | G | -0.0261729 | 0.00444286 | 3.83398e-09 | -0.00623534 | 0.0219954 | 0.776805 |
| rs10476484 | G | A | 0.0149229 | 0.00256362 | 5.85194e-09 | -0.00325981 | 0.0138006 | 0.813271 |
| rs10497655 | C | T | -0.0151713 | 0.0024705 | 8.18502e-10 | -0.0148666 | 0.0134491 | 0.268989 |
| rs10782302 | C | T | 0.0126181 | 0.0022988 | 4.04604e-08 | 0.0232977 | 0.0131379 | 0.0761763 |
| rs10811883 | T | C | -0.01725 | 0.00241597 | 9.31751e-13 | 0.0102227 | 0.0135691 | 0.451224 |
| rs10935181 | A | G | -0.0165427 | 0.00232243 | 1.05803e-12 | 0.0115049 | 0.0131976 | 0.383352 |
| rs11039182 | C | T | 0.0196363 | 0.00258543 | 3.08319e-14 | -0.010707 | 0.0168965 | 0.526289 |
| rs11082011 | T | C | -0.0228736 | 0.00242768 | 4.43813e-21 | -0.0322483 | 0.0148952 | 0.0303865 |
| rs11090045 | A | G | 0.0192599 | 0.0025466 | 3.93097e-14 | 0.0207176 | 0.0138723 | 0.135319 |
| rs11184985 | C | T | -0.0142811 | 0.00258668 | 3.36899e-08 | -0.0110261 | 0.015121 | 0.465884 |
| rs11263943 | G | A | -0.0157982 | 0.00232943 | 1.18796e-11 | -0.0124599 | 0.01331 | 0.349205 |
| rs11509880 | A | G | 0.0156797 | 0.00241822 | 8.91661e-11 | -0.000683166 | 0.0140663 | 0.961264 |
| rs11608355 | C | T | 0.019565 | 0.00247157 | 2.44287e-15 | 0.0122564 | 0.0156479 | 0.433475 |
| rs11627348 | A | C | 0.0187324 | 0.0032275 | 6.47694e-09 | -0.00155334 | 0.0208765 | 0.940687 |
| rs11759026 | G | A | -0.0150881 | 0.0027408 | 3.68104e-08 | -0.00941212 | 0.0152366 | 0.536754 |
| rs11975 | C | A | 0.0216038 | 0.0032738 | 4.13809e-11 | 0.0167493 | 0.0191684 | 0.38223 |
| rs12092768 | A | G | -0.0153096 | 0.00254524 | 1.79498e-09 | -0.00814394 | 0.0145643 | 0.576046 |
| rs12137398 | T | C | 0.0178531 | 0.00292386 | 1.022e-09 | -0.0189968 | 0.016803 | 0.258239 |
| rs1282545 | C | T | 0.0167823 | 0.00234816 | 8.83894e-13 | 0.00621034 | 0.0131989 | 0.637984 |
| rs12902680 | C | T | 0.0134144 | 0.00238139 | 1.77301e-08 | -0.0166416 | 0.0143028 | 0.244617 |
| rs12903078 | A | G | 0.0128833 | 0.00233731 | 3.53997e-08 | -0.0166993 | 0.0134531 | 0.214494 |
| rs13226841 | C | T | 0.017193 | 0.00229669 | 7.12525e-14 | 0.0144165 | 0.0131835 | 0.274161 |
| rs1422192 | A | G | 0.0190464 | 0.00308544 | 6.68005e-10 | -0.025892 | 0.0203253 | 0.202707 |
| rs1542212 | G | T | 0.0159476 | 0.00236156 | 1.44611e-11 | -0.00937493 | 0.0142454 | 0.510472 |
| rs16854051 | T | C | -0.0156286 | 0.00283847 | 3.66201e-08 | -0.0135 | 0.0167864 | 0.421268 |
| rs17096778 | G | A | -0.0320548 | 0.00554869 | 7.60309e-09 | -0.0488125 | 0.0468205 | 0.297159 |
| rs17487484 | G | T | 0.0142497 | 0.0022939 | 5.23998e-10 | -0.00271536 | 0.0131841 | 0.836825 |
| rs17619012 | T | G | -0.0156225 | 0.00279422 | 2.26001e-08 | -0.0160023 | 0.0139811 | 0.252388 |
| rs1892350 | G | A | 0.0141816 | 0.00229997 | 6.99906e-10 | 0.00559797 | 0.0131857 | 0.671165 |
| rs2149351 | G | T | -0.0186461 | 0.00270194 | 5.14991e-12 | -0.00380733 | 0.0171511 | 0.824324 |
| rs2269426 | A | G | 0.0184312 | 0.00239025 | 1.25112e-14 | -0.0239779 | 0.0133137 | 0.0717034 |
| rs2295094 | A | G | 0.0172829 | 0.00303849 | 1.28499e-08 | -0.00645306 | 0.0215709 | 0.764821 |
| rs2488401 | T | C | -0.0172249 | 0.00285795 | 1.67101e-09 | -0.0251235 | 0.0188485 | 0.182559 |
| rs2503775 | G | A | -0.021811 | 0.0034 | 1.40799e-10 | 0.000284219 | 0.021251 | 0.989329 |
| rs2717043 | T | C | 0.0151639 | 0.00238015 | 1.87499e-10 | 0.026797 | 0.0135884 | 0.0486038 |
| rs28986304 | C | T | 0.0212038 | 0.00345114 | 8.04693e-10 | 0.0158264 | 0.027268 | 0.561643 |
| rs297346 | G | A | -0.0155813 | 0.00238611 | 6.56145e-11 | 0.0136894 | 0.0152167 | 0.368317 |
| rs3124426 | T | C | -0.0153159 | 0.00272379 | 1.87802e-08 | -0.0237683 | 0.0161477 | 0.141039 |
| rs34979551 | G | A | -0.0189387 | 0.00343466 | 3.50203e-08 | -0.0345925 | 0.0226911 | 0.127385 |
| rs35982947 | C | A | -0.0136214 | 0.00240703 | 1.521e-08 | -0.0104907 | 0.0141279 | 0.457755 |
| rs36006259 | T | C | -0.0179004 | 0.00320796 | 2.40902e-08 | 0.00915284 | 0.016561 | 0.580485 |
| rs3936093 | G | A | 0.0148324 | 0.00233104 | 1.97702e-10 | 0.0200321 | 0.0132792 | 0.131419 |
| rs4245154 | G | A | -0.0196045 | 0.00232833 | 3.75319e-17 | -0.0269569 | 0.0150786 | 0.0738142 |
| rs4362360 | C | T | -0.0126458 | 0.00229465 | 3.56903e-08 | -0.00460809 | 0.013326 | 0.729496 |
| rs4396680 | G | A | -0.0167747 | 0.00285091 | 3.99604e-09 | -0.054649 | 0.0213708 | 0.0105524 |
| rs4530683 | G | A | 0.0129193 | 0.00234131 | 3.42902e-08 | 0.0270132 | 0.0132836 | 0.0419952 |
| rs4578918 | C | T | -0.0169464 | 0.00263183 | 1.20401e-10 | -0.00480849 | 0.0147568 | 0.744538 |
| rs4585149 | C | T | 0.0175304 | 0.00302927 | 7.16292e-09 | 0.00636755 | 0.0160931 | 0.692349 |
| rs56084168 | T | C | -0.0232368 | 0.00323453 | 6.78422e-13 | -0.00848229 | 0.0169496 | 0.616763 |
| rs58007470 | T | C | 0.0154904 | 0.00279509 | 2.99199e-08 | 0.0118337 | 0.0146412 | 0.418948 |
| rs59491086 | G | A | -0.0188454 | 0.00284072 | 3.26964e-11 | -0.0284871 | 0.0152465 | 0.0617007 |
| rs62062288 | A | G | 0.0334444 | 0.00284246 | 5.82371e-32 | -0.0220912 | 0.024243 | 0.362169 |
| rs62550480 | T | C | -0.0181085 | 0.00279539 | 9.31537e-11 | 0.0398638 | 0.0175707 | 0.0232825 |
| rs6601444 | T | C | 0.0162582 | 0.00289394 | 1.935e-08 | -0.00482417 | 0.0167762 | 0.773683 |
| rs6737187 | A | G | -0.0141254 | 0.00250405 | 1.68601e-08 | -0.00758061 | 0.0143521 | 0.597369 |
| rs674437 | A | G | -0.0145107 | 0.00229854 | 2.73502e-10 | -0.00806749 | 0.0133002 | 0.544135 |
| rs6970541 | G | T | -0.0134835 | 0.00231159 | 5.45406e-09 | -0.00901199 | 0.0131398 | 0.492806 |
| rs6997840 | C | T | 0.0131099 | 0.00237197 | 3.249e-08 | 0.00242604 | 0.0131482 | 0.85361 |
| rs7033345 | C | T | -0.0153362 | 0.00256931 | 2.38298e-09 | -0.0043777 | 0.0138713 | 0.75231 |
| rs72759273 | G | A | -0.0158225 | 0.00289789 | 4.76102e-08 | -0.0174533 | 0.015676 | 0.265547 |
| rs7338774 | G | A | 0.0148001 | 0.00246217 | 1.84599e-09 | 0.0257445 | 0.0142694 | 0.0712033 |
| rs75614054 | T | C | 0.0311936 | 0.00409312 | 2.51826e-14 | 0.0144687 | 0.0212624 | 0.496199 |
| rs76082995 | C | T | 0.0135575 | 0.00232867 | 5.80604e-09 | -0.0214339 | 0.0131799 | 0.103894 |
| rs77580167 | T | G | -0.018192 | 0.00330402 | 3.67697e-08 | -0.00132999 | 0.0205281 | 0.948342 |
| rs7837935 | G | T | 0.0175613 | 0.00313315 | 2.082e-08 | -0.00995268 | 0.0201458 | 0.621282 |
| rs7895261 | G | T | -0.0142086 | 0.00254361 | 2.32702e-08 | -0.0059118 | 0.0158918 | 0.709892 |
| rs802425 | T | C | -0.0131476 | 0.00231472 | 1.344e-08 | -0.0105199 | 0.0134211 | 0.43314 |
| rs860626 | G | T | -0.0152341 | 0.00254198 | 2.05902e-09 | -0.0030367 | 0.013608 | 0.823415 |
| rs9881798 | C | A | 0.015771 | 0.0023406 | 1.60214e-11 | 0.01096 | 0.0135555 | 0.418788 |
| rs988867 | T | G | 0.0183242 | 0.00326519 | 1.99701e-08 | -0.0401392 | 0.0188675 | 0.0333849 |
| rs998884 | G | A | 0.0177656 | 0.00235994 | 5.15941e-14 | -0.00782256 | 0.0131622 | 0.552299 |

| **Supplementary Table 4.** Genetic instrumental variables used for two-sample MR analyses for Worry and probable sleep bruxism. EA=effect allele, OA=other allele | | | | | | | | |
| --- | --- | --- | --- | --- | --- | --- | --- | --- |
|  |  |  |  |  |  |  |  |  |
|  |  |  | **Exposure (Worry)** | | | **Outcome (Probable Sleep Bruxism)** | | |
| **Variant** | **EA** | **OA** | **Beta** | **SE** | **P-value** | **Beta** | **SE** | **P-value** |
| rs10005233 | T | C | 0.01508 | 0.002386 | 2.629e-10 | -0.0283521 | 0.013227 | 0.032073 |
| rs10034259 | C | A | -0.0187 | 0.002996 | 4.32195e-10 | -0.00256296 | 0.0161987 | 0.874283 |
| rs10871777 | G | A | -0.02024 | 0.0028 | 4.89328e-13 | -0.0190069 | 0.0168245 | 0.258595 |
| rs11090045 | A | G | 0.01788 | 0.002643 | 1.33506e-11 | 0.0207176 | 0.0138723 | 0.135319 |
| rs11204421 | C | T | 0.01322 | 0.002409 | 4.03701e-08 | 0.01121 | 0.0134821 | 0.405707 |
| rs112591851 | T | C | -0.03774 | 0.006898 | 4.47003e-08 | -0.0319817 | 0.0327746 | 0.329161 |
| rs13262595 | G | A | -0.01744 | 0.002394 | 3.22626e-13 | -0.013681 | 0.0133808 | 0.306575 |
| rs1330745 | C | A | -0.01819 | 0.002893 | 3.19897e-10 | -0.00246743 | 0.0156322 | 0.87458 |
| rs13324323 | T | C | -0.01739 | 0.002912 | 2.355e-09 | 0.00481676 | 0.0148512 | 0.745686 |
| rs13328187 | C | T | 0.01711 | 0.002728 | 3.59898e-10 | -0.00990394 | 0.0172213 | 0.565226 |
| rs1593304 | G | A | 0.01679 | 0.002993 | 2.048e-08 | 0.0186155 | 0.0199594 | 0.350991 |
| rs17196295 | G | A | 0.01563 | 0.002637 | 3.05302e-09 | 0.019523 | 0.013658 | 0.152882 |
| rs1724725 | G | A | 0.02748 | 0.004798 | 1.02301e-08 | 0.0173782 | 0.0323888 | 0.591578 |
| rs17532098 | T | C | 0.02318 | 0.003895 | 2.64198e-09 | 0.0304554 | 0.0204676 | 0.136755 |
| rs1826787 | T | C | -0.02258 | 0.003781 | 2.34099e-09 | -0.00859923 | 0.0198255 | 0.664474 |
| rs1890184 | C | A | -0.01785 | 0.002439 | 2.48085e-13 | 0.00340864 | 0.0135148 | 0.800875 |
| rs1998122 | A | G | -0.01403 | 0.00245 | 1.02601e-08 | -0.0340075 | 0.0133644 | 0.0109391 |
| rs2269426 | A | G | 0.01871 | 0.002478 | 4.30824e-14 | -0.0239779 | 0.0133137 | 0.0717034 |
| rs2488401 | T | C | -0.01782 | 0.003008 | 3.14297e-09 | -0.0251235 | 0.0188485 | 0.182559 |
| rs2672852 | T | C | 0.0137 | 0.002382 | 8.82795e-09 | 0.0108177 | 0.0132123 | 0.412922 |
| rs3026401 | T | C | -0.01945 | 0.00295 | 4.30527e-11 | -0.00606314 | 0.0149637 | 0.685338 |
| rs34644694 | A | C | 0.01577 | 0.002669 | 3.404e-09 | 0.00510992 | 0.0145465 | 0.725376 |
| rs353547 | C | T | 0.01697 | 0.002456 | 4.80729e-12 | 0.031761 | 0.0132249 | 0.0163234 |
| rs3742020 | C | T | 0.01627 | 0.00257 | 2.42198e-10 | 0.012297 | 0.0156484 | 0.431969 |
| rs3751855 | C | T | -0.01391 | 0.002445 | 1.276e-08 | -0.00207168 | 0.0133931 | 0.877071 |
| rs391236 | G | A | -0.01494 | 0.002599 | 8.99104e-09 | -0.0280152 | 0.0139782 | 0.0450474 |
| rs4245150 | T | G | 0.01484 | 0.00244 | 1.17201e-09 | 0.0200924 | 0.0162143 | 0.215282 |
| rs45536634 | A | G | 0.02428 | 0.003764 | 1.104e-10 | 0.0205289 | 0.0191873 | 0.284654 |
| rs4684833 | T | C | -0.01563 | 0.002866 | 4.97004e-08 | 0.0361144 | 0.0160164 | 0.0241435 |
| rs480330 | T | C | -0.01695 | 0.002509 | 1.40896e-11 | 0.00645687 | 0.0136262 | 0.6356 |
| rs502652 | T | C | -0.01357 | 0.002464 | 3.63898e-08 | 0.00171897 | 0.0135842 | 0.899303 |
| rs56084168 | T | C | -0.02129 | 0.003354 | 2.20602e-10 | -0.00848229 | 0.0169496 | 0.616763 |
| rs56133711 | A | G | -0.01517 | 0.002722 | 2.52302e-08 | -0.0314752 | 0.0151853 | 0.0381971 |
| rs57360718 | C | T | 0.02831 | 0.004882 | 6.69098e-09 | -0.0152675 | 0.0280245 | 0.585898 |
| rs60642411 | A | G | 0.019 | 0.003481 | 4.82703e-08 | 0.00537768 | 0.0208362 | 0.796335 |
| rs61731122 | A | G | -0.04219 | 0.007038 | 2.04301e-09 | -0.0283106 | 0.046235 | 0.540326 |
| rs62081501 | A | G | 0.02984 | 0.004355 | 7.30466e-12 | 0.0476286 | 0.0233488 | 0.0413638 |
| rs62551581 | C | A | -0.01613 | 0.002884 | 2.225e-08 | 0.0383326 | 0.0174831 | 0.02834 |
| rs6478623 | G | T | 0.01539 | 0.002694 | 1.11699e-08 | 0.0144544 | 0.0137802 | 0.294213 |
| rs6807666 | C | A | 0.02257 | 0.00249 | 1.26794e-19 | -0.00308655 | 0.0147071 | 0.833771 |
| rs7033345 | C | T | -0.01547 | 0.002679 | 7.70495e-09 | -0.0043777 | 0.0138713 | 0.75231 |
| rs7152906 | C | T | 0.01765 | 0.002379 | 1.17896e-13 | -0.00344824 | 0.0131308 | 0.792854 |
| rs7207400 | C | T | 0.02119 | 0.002676 | 2.38397e-15 | -0.00863978 | 0.0200901 | 0.667158 |
| rs75195552 | A | G | 0.01678 | 0.002891 | 6.51899e-09 | -0.0323445 | 0.0149214 | 0.0301842 |
| rs75614054 | T | C | 0.02406 | 0.004207 | 1.06699e-08 | 0.0144687 | 0.0212624 | 0.496199 |
| rs7567451 | T | G | 0.01589 | 0.002689 | 3.474e-09 | 0.0286126 | 0.0149449 | 0.0555508 |
| rs78260322 | G | A | 0.02154 | 0.00385 | 2.20999e-08 | 0.0382136 | 0.0244889 | 0.118654 |
| rs79827531 | A | G | 0.01773 | 0.002993 | 3.16497e-09 | -0.0354675 | 0.0167986 | 0.034744 |
| rs998884 | G | A | 0.01547 | 0.002453 | 2.877e-10 | -0.00782256 | 0.0131622 | 0.552299 |

| **Supplementary Table 5.** Genetic instrumental variables used for two-sample MR analyses for Depressed Affect and probable sleep bruxism. EA=effect allele, OA=other allele | | | | | | | | |
| --- | --- | --- | --- | --- | --- | --- | --- | --- |
|  |  |  |  |  |  |  |  |  |
|  |  |  | **Exposure (Depressed Affect)** | | | **Outcome (Probable Sleep Bruxism)** | | |
| **Variant** | **EA** | **OA** | **Beta** | **SE** | **P-value** | **Beta** | **SE** | **P-value** |
| rs10020288 | A | G | -0.01346 | 0.002416 | 2.56301e-08 | 0.0294467 | 0.0137493 | 0.0322188 |
| rs10144845 | T | C | 0.01604 | 0.002483 | 1.05099e-10 | 0.0440137 | 0.0134845 | 0.00109842 |
| rs10950393 | C | T | 0.01561 | 0.002336 | 2.37192e-11 | 0.0019154 | 0.0135881 | 0.8879 |
| rs11039149 | G | A | 0.01936 | 0.002579 | 6.00759e-14 | -0.0105806 | 0.0168931 | 0.5311 |
| rs11209175 | T | C | -0.01453 | 0.00241 | 1.64502e-09 | -0.00757284 | 0.0131646 | 0.565128 |
| rs11599236 | C | T | -0.01585 | 0.00239 | 3.30522e-11 | 0.00151224 | 0.0131659 | 0.908556 |
| rs11605020 | A | G | 0.01387 | 0.002351 | 3.59203e-09 | -0.0142519 | 0.0134279 | 0.288525 |
| rs11608355 | C | T | 0.01457 | 0.002494 | 5.10199e-09 | 0.0122564 | 0.0156479 | 0.433475 |
| rs11693031 | G | A | -0.01573 | 0.002524 | 4.58596e-10 | 0.00385986 | 0.0140653 | 0.78376 |
| rs12967855 | G | A | -0.01741 | 0.002478 | 2.10911e-12 | -0.0323603 | 0.0148947 | 0.0298099 |
| rs13122395 | A | G | 0.01356 | 0.002461 | 3.63396e-08 | -0.0242425 | 0.0153598 | 0.114495 |
| rs1422192 | A | G | 0.01917 | 0.003168 | 1.44202e-09 | -0.025892 | 0.0203253 | 0.202707 |
| rs1542212 | G | T | 0.0133 | 0.002381 | 2.31302e-08 | -0.00937493 | 0.0142454 | 0.510472 |
| rs17432675 | C | T | -0.01373 | 0.002502 | 4.04203e-08 | -0.00946659 | 0.013745 | 0.490992 |
| rs1782179 | C | T | 0.01471 | 0.002597 | 1.5e-08 | 0.0215405 | 0.0143087 | 0.132219 |
| rs2042555 | A | G | 0.0144 | 0.002354 | 9.45301e-10 | -0.0092087 | 0.0131491 | 0.483721 |
| rs209156 | A | C | 0.02831 | 0.005157 | 4.02903e-08 | -0.0403855 | 0.0353488 | 0.253253 |
| rs2149351 | G | T | -0.0162 | 0.002711 | 2.273e-09 | -0.00380733 | 0.0171511 | 0.824324 |
| rs2396133 | G | A | 0.01326 | 0.002325 | 1.17601e-08 | 0.00290074 | 0.013204 | 0.826115 |
| rs2717043 | T | C | 0.01528 | 0.00239 | 1.637e-10 | 0.026797 | 0.0135884 | 0.0486038 |
| rs28893517 | G | A | 0.02848 | 0.004583 | 5.15798e-10 | 0.0187274 | 0.0410436 | 0.64819 |
| rs2895249 | A | G | -0.0133 | 0.002381 | 2.339e-08 | -0.00765902 | 0.0133545 | 0.566295 |
| rs297346 | G | A | -0.0162 | 0.00241 | 1.80717e-11 | 0.0136894 | 0.0152167 | 0.368317 |
| rs35738585 | G | T | -0.01722 | 0.002337 | 1.72982e-13 | -0.0269823 | 0.0150765 | 0.0735038 |
| rs35755513 | T | C | 0.02414 | 0.0044 | 4.11803e-08 | -0.0586179 | 0.0274769 | 0.0328958 |
| rs3795310 | T | C | 0.01376 | 0.002325 | 3.24101e-09 | 0.0478218 | 0.0136567 | 0.000462296 |
| rs4578918 | C | T | -0.01792 | 0.002634 | 1.02896e-11 | -0.00480849 | 0.0147568 | 0.744538 |
| rs4625 | G | A | -0.01564 | 0.002503 | 4.20901e-10 | 0.00803122 | 0.0132746 | 0.545174 |
| rs4632195 | T | C | 0.0159 | 0.002316 | 6.62522e-12 | -0.00972242 | 0.0131564 | 0.459913 |
| rs55965054 | T | C | -0.01391 | 0.002337 | 2.67098e-09 | -0.0141466 | 0.0131626 | 0.282485 |
| rs59382200 | G | A | -0.01581 | 0.002368 | 2.44118e-11 | -0.0193831 | 0.0131934 | 0.141793 |
| rs599550 | A | G | 0.02725 | 0.003235 | 3.69063e-17 | 0.00462362 | 0.0195057 | 0.812626 |
| rs60393230 | G | A | 0.01397 | 0.002366 | 3.56903e-09 | 0.0289883 | 0.0135262 | 0.0321033 |
| rs62172117 | A | G | -0.01405 | 0.002417 | 6.188e-09 | 0.0215571 | 0.0140236 | 0.124244 |
| rs6795372 | G | T | 0.0145 | 0.002393 | 1.39499e-09 | -0.000178969 | 0.01433 | 0.990035 |
| rs6818081 | T | C | 0.01805 | 0.003251 | 2.828e-08 | 0.0216977 | 0.0182773 | 0.235173 |
| rs6900114 | A | G | -0.01464 | 0.002621 | 2.30001e-08 | -0.0154578 | 0.0148926 | 0.29929 |
| rs7175083 | C | T | -0.01467 | 0.002311 | 2.21197e-10 | -0.00646502 | 0.0131921 | 0.624087 |
| rs721496 | A | G | 0.01561 | 0.002663 | 4.59695e-09 | -0.00305237 | 0.0141385 | 0.829072 |
| rs7502590 | G | A | -0.01801 | 0.003254 | 3.11803e-08 | -0.00978309 | 0.0169759 | 0.564417 |
| rs75650221 | T | C | -0.03572 | 0.006026 | 3.07503e-09 | -0.0220129 | 0.0377204 | 0.559504 |
| rs76923064 | C | T | 0.05634 | 0.01019 | 3.24399e-08 | -0.052299 | 0.0443188 | 0.237975 |
| rs77087420 | G | A | -0.03284 | 0.005085 | 1.05599e-10 | -0.0311001 | 0.0306149 | 0.309702 |
| rs836927 | A | C | 0.01484 | 0.002374 | 4.10601e-10 | 0.00605862 | 0.0131962 | 0.646149 |
| rs9852417 | A | C | -0.0131 | 0.002361 | 2.83903e-08 | -0.0295856 | 0.0133604 | 0.0267991 |
| rs9858071 | C | T | 0.01607 | 0.002556 | 3.24997e-10 | 0.00903923 | 0.0144701 | 0.532179 |
| rs9930139 | C | A | 0.01351 | 0.002369 | 1.19001e-08 | -0.00105125 | 0.0133622 | 0.937293 |

| **Supplementary Table 6.** Genetic instrumental variables used for two-sample MR analyses of SESA and probable sleep bruxism. EA=effect allele, OA=other allele | | | | | | | | |
| --- | --- | --- | --- | --- | --- | --- | --- | --- |
|  |  |  |  |  |  |  |  |  |
|  |  |  | **Exposure (SESA)** | | | **Outcome (Probable Sleep Bruxism)** | | |
| **Variant** | **EA** | **OA** | **Beta** | **SE** | **P-value** | **Beta** | **SE** | **P-value** |
| rs10119773 | A | G | -0.01576 | 0.002387 | 4.1e-11 | -0.0095673 | 0.0131999 | 0.468574 |
| rs10476484 | G | A | 0.01476 | 0.00263 | 1.98e-08 | -0.00325981 | 0.0138006 | 0.813271 |
| rs10765820 | A | G | 0.01406 | 0.002335 | 1.756e-09 | 0.00788061 | 0.0133135 | 0.553898 |
| rs11022762 | T | C | 0.01662 | 0.002394 | 3.832e-12 | 0.0093332 | 0.0132059 | 0.479725 |
| rs11090045 | A | G | 0.01507 | 0.002585 | 5.565e-09 | 0.0207176 | 0.0138723 | 0.135319 |
| rs11115117 | C | T | 0.01946 | 0.003474 | 2.131e-08 | -0.0046588 | 0.0200761 | 0.816493 |
| rs11167957 | C | T | -0.01324 | 0.002414 | 4.129e-08 | -0.017526 | 0.0133135 | 0.188035 |
| rs11263943 | A | G | 0.01615 | 0.002363 | 8.229e-12 | 0.0124599 | 0.01331 | 0.349205 |
| rs11613183 | C | T | 0.02542 | 0.004531 | 2.015e-08 | 0.0436881 | 0.0234862 | 0.0628623 |
| rs11627348 | A | C | 0.01887 | 0.003297 | 1.054e-08 | -0.00155334 | 0.0208765 | 0.940687 |
| rs11665070 | G | A | 0.02149 | 0.00248 | 4.517e-18 | 0.0329653 | 0.0148718 | 0.0266477 |
| rs1282545 | C | T | 0.0139 | 0.002391 | 6.119e-09 | 0.00621034 | 0.0131989 | 0.637984 |
| rs13070517 | T | G | -0.01362 | 0.002417 | 1.739e-08 | -0.0260722 | 0.0136768 | 0.0566096 |
| rs17049509 | T | C | -0.01456 | 0.002564 | 1.359e-08 | -0.0478666 | 0.0148701 | 0.00128644 |
| rs1941355 | C | A | 0.01378 | 0.002428 | 1.375e-08 | -0.0156774 | 0.013203 | 0.235068 |
| rs1978573 | T | C | -0.01454 | 0.002558 | 1.312e-08 | -0.0157643 | 0.0135015 | 0.24297 |
| rs2282040 | G | A | 0.02858 | 0.003988 | 7.712e-13 | 0.0149461 | 0.0212037 | 0.480884 |
| rs2305411 | C | T | -0.01549 | 0.002634 | 4.044e-09 | -0.00246987 | 0.0158603 | 0.876249 |
| rs2627019 | T | C | 0.01292 | 0.002355 | 4.073e-08 | -0.00535517 | 0.0131457 | 0.683737 |
| rs3124426 | C | T | 0.01577 | 0.002809 | 1.972e-08 | 0.0237683 | 0.0161477 | 0.141039 |
| rs314289 | C | T | -0.01296 | 0.002352 | 3.631e-08 | -0.0151798 | 0.0133479 | 0.255437 |
| rs34351088 | C | T | 0.01946 | 0.002328 | 6.219e-17 | 0.0143574 | 0.0131848 | 0.27618 |
| rs34657012 | A | C | 0.01532 | 0.002589 | 3.299e-09 | -0.0121352 | 0.014577 | 0.405132 |
| rs35393419 | T | C | 0.01882 | 0.002686 | 2.468e-12 | 0.0198134 | 0.0175219 | 0.258149 |
| rs35738585 | G | T | -0.01911 | 0.002356 | 4.944e-16 | -0.0269823 | 0.0150765 | 0.0735038 |
| rs3772882 | A | C | -0.01465 | 0.002408 | 1.178e-09 | -0.00349559 | 0.0131784 | 0.790815 |
| rs391957 | T | C | -0.01506 | 0.002379 | 2.443e-10 | -0.0151138 | 0.0149277 | 0.311314 |
| rs45601232 | G | A | 0.06412 | 0.01088 | 3.773e-09 | 0.0106771 | 0.10591 | 0.919699 |
| rs4791331 | T | C | -0.0139 | 0.00235 | 3.318e-09 | -0.00869565 | 0.0131844 | 0.50955 |
| rs527528 | T | C | 0.01856 | 0.002475 | 6.566e-14 | -0.00873687 | 0.0135763 | 0.519874 |
| rs56084168 | T | C | -0.01968 | 0.003283 | 2.025e-09 | -0.00848229 | 0.0169496 | 0.616763 |
| rs62442206 | C | A | -0.02117 | 0.003454 | 8.871e-10 | 0.00634043 | 0.0190064 | 0.738686 |
| rs6439649 | G | T | -0.01432 | 0.002375 | 1.668e-09 | 0.0125597 | 0.0131717 | 0.340316 |
| rs6490177 | A | C | 0.01863 | 0.003054 | 1.064e-09 | -0.00566998 | 0.0171132 | 0.740401 |
| rs6941112 | A | G | 0.01765 | 0.002566 | 6.088e-12 | -0.0255701 | 0.0136586 | 0.0611942 |
| rs7567451 | G | T | -0.0144 | 0.002632 | 4.541e-08 | -0.0286126 | 0.0149449 | 0.0555508 |
| rs75737648 | T | C | -0.02096 | 0.003712 | 1.637e-08 | -0.0163912 | 0.0239226 | 0.493232 |
| rs761898 | A | G | -0.01362 | 0.002443 | 2.48e-08 | 0.0100182 | 0.013645 | 0.462827 |
| rs80143279 | C | T | 0.02682 | 0.002794 | 8.055e-22 | -0.024651 | 0.0239716 | 0.303789 |
| rs9366697 | C | T | -0.01363 | 0.002432 | 2.073e-08 | -0.0278951 | 0.0136532 | 0.0410412 |
| rs9462364 | A | G | -0.01348 | 0.002342 | 8.589e-09 | 0.00123243 | 0.0133473 | 0.926432 |
| rs9811585 | G | T | 0.01418 | 0.002404 | 3.688e-09 | -0.015257 | 0.0144072 | 0.289608 |

**Neuroticism**


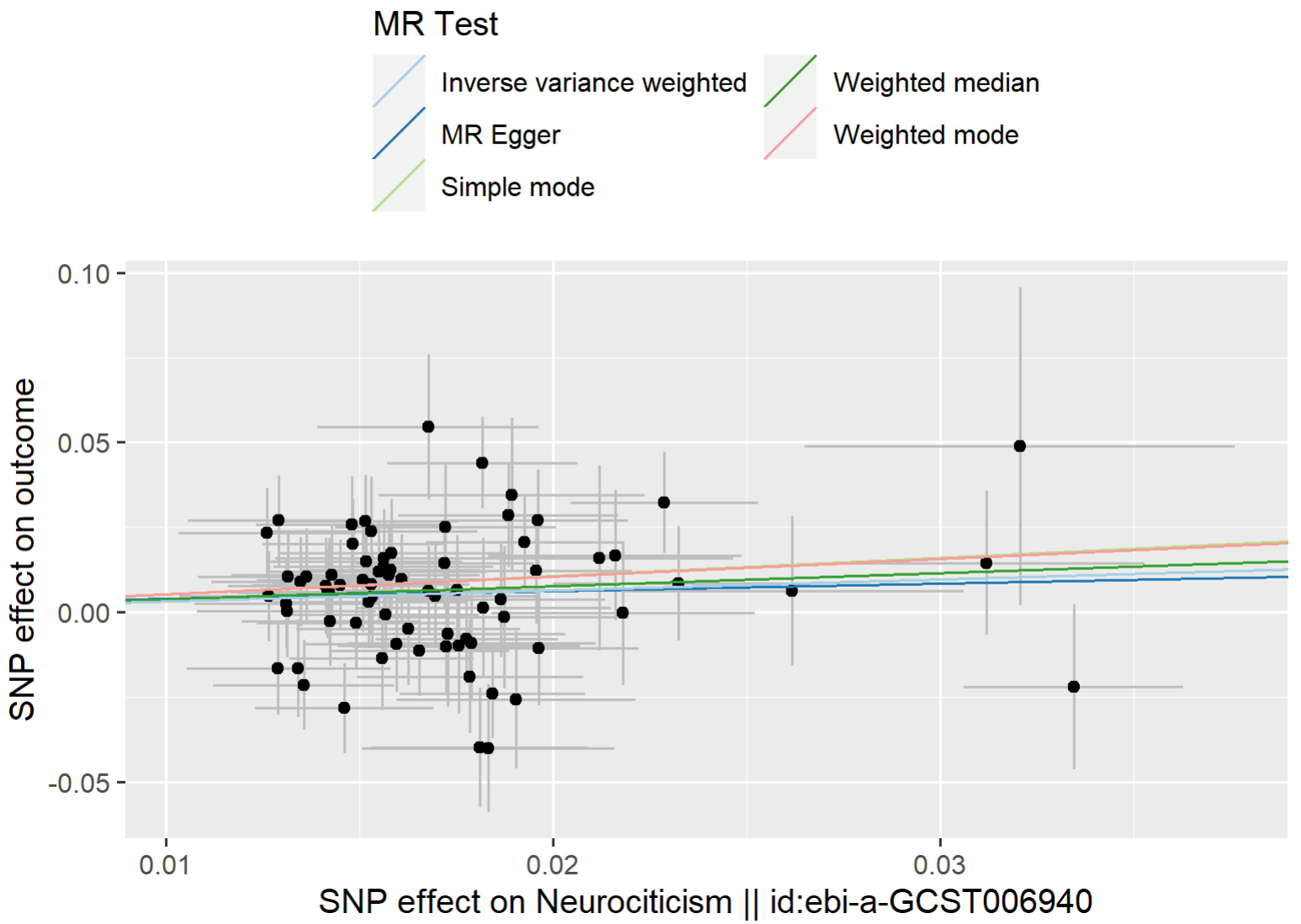


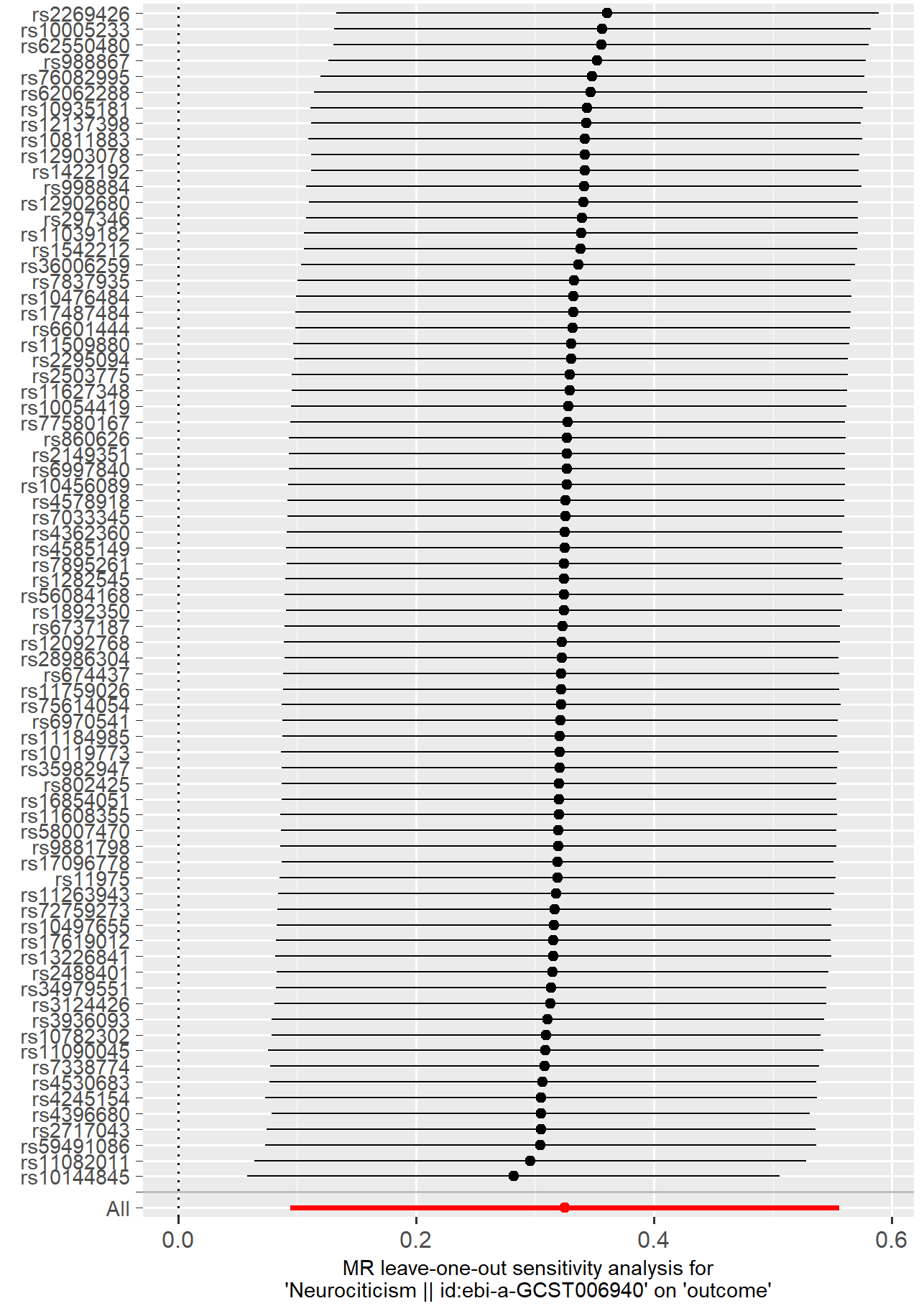


**Supplementary Figure 1.** Scatter plot with lines of different regression analyses of genetic associations (top), and a leave one out analysis (bottom) for inverse variance weighted (IVW) Mendelian randomization estimate, of neuroticism score on probable sleep bruxism.

MR = Mendelian randomization; SNP = single nucleotide polymorphism; rs = reference SNP (ID) cluster


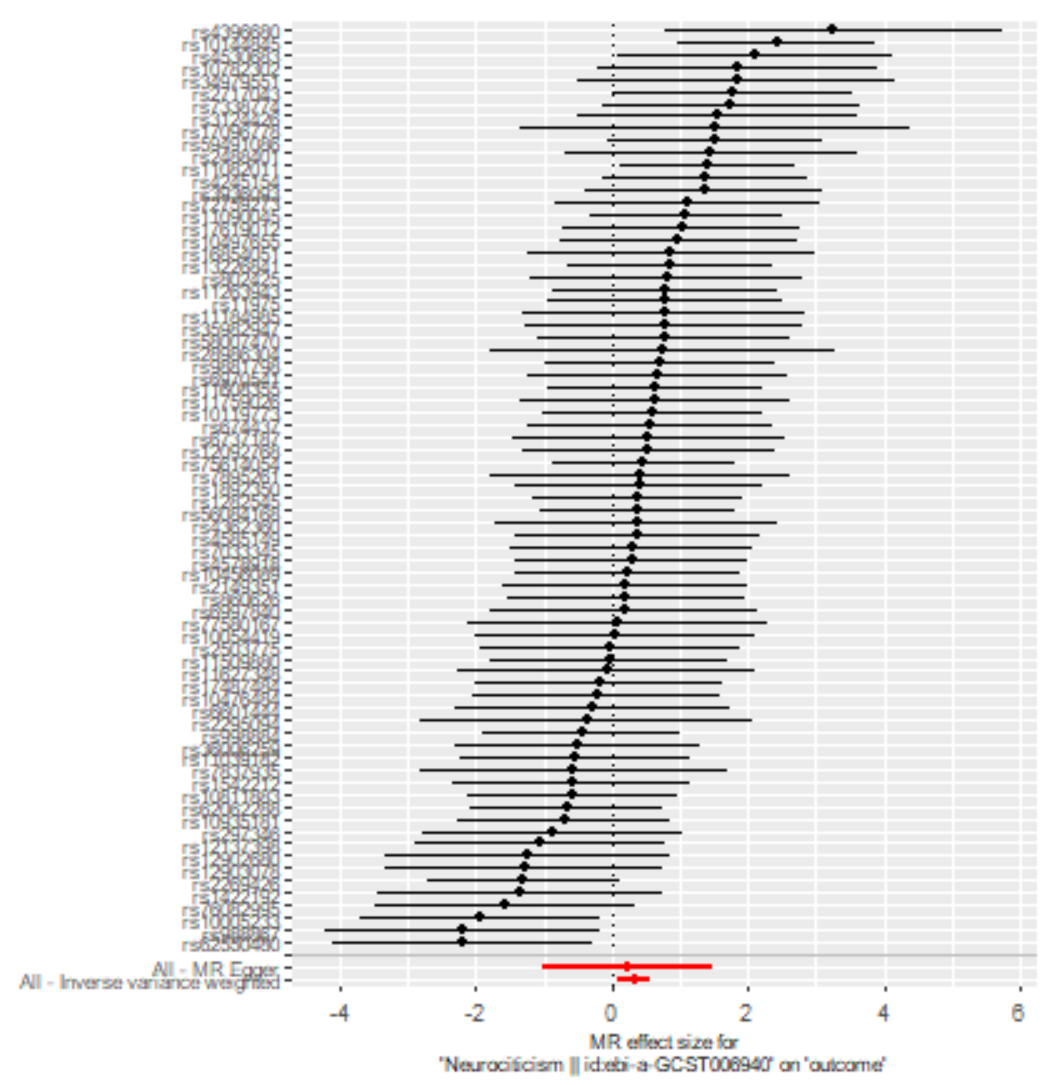


**Supplementary Figure 2.** Forest plot of single single nucleotide polymorphism (SNP) Mendelian randimization (MR) analysis depicting the relationship between neuroticism scores (exposure) and probable sleep bruxism (outcome) with 75 valid SNPs. Black points show the causal effect estimate (beta coefficient or logOR) and the black line indicates the 95% CI of the estimate. The red points and lines show the causal effect estimate using the MR Egger or the inverse variance weighted (IVW) method and 95% CI, respectively.

OR = odds ratio; CI = confidence interval; rs = reference SNP (ID) cluster

**Worry**


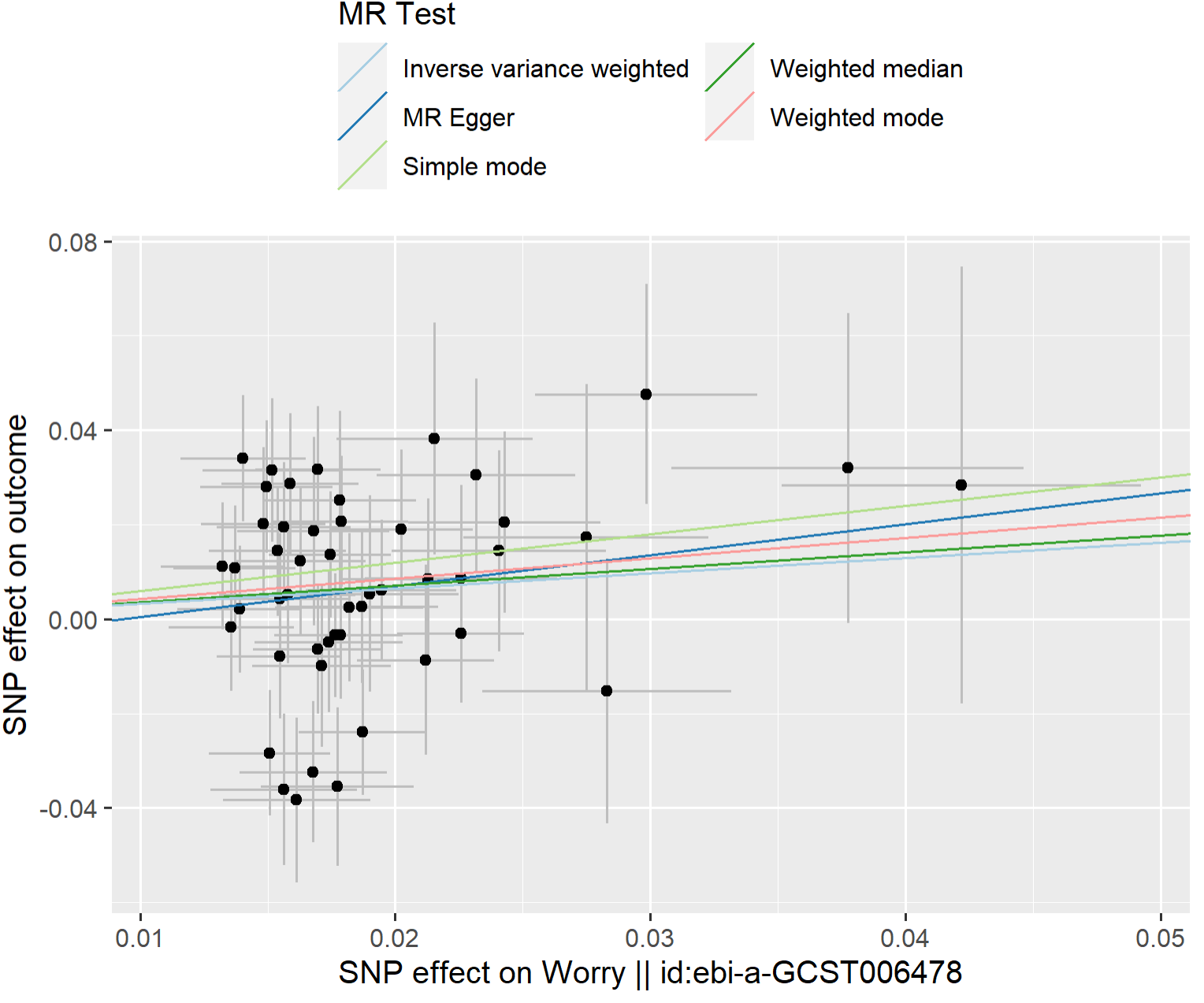


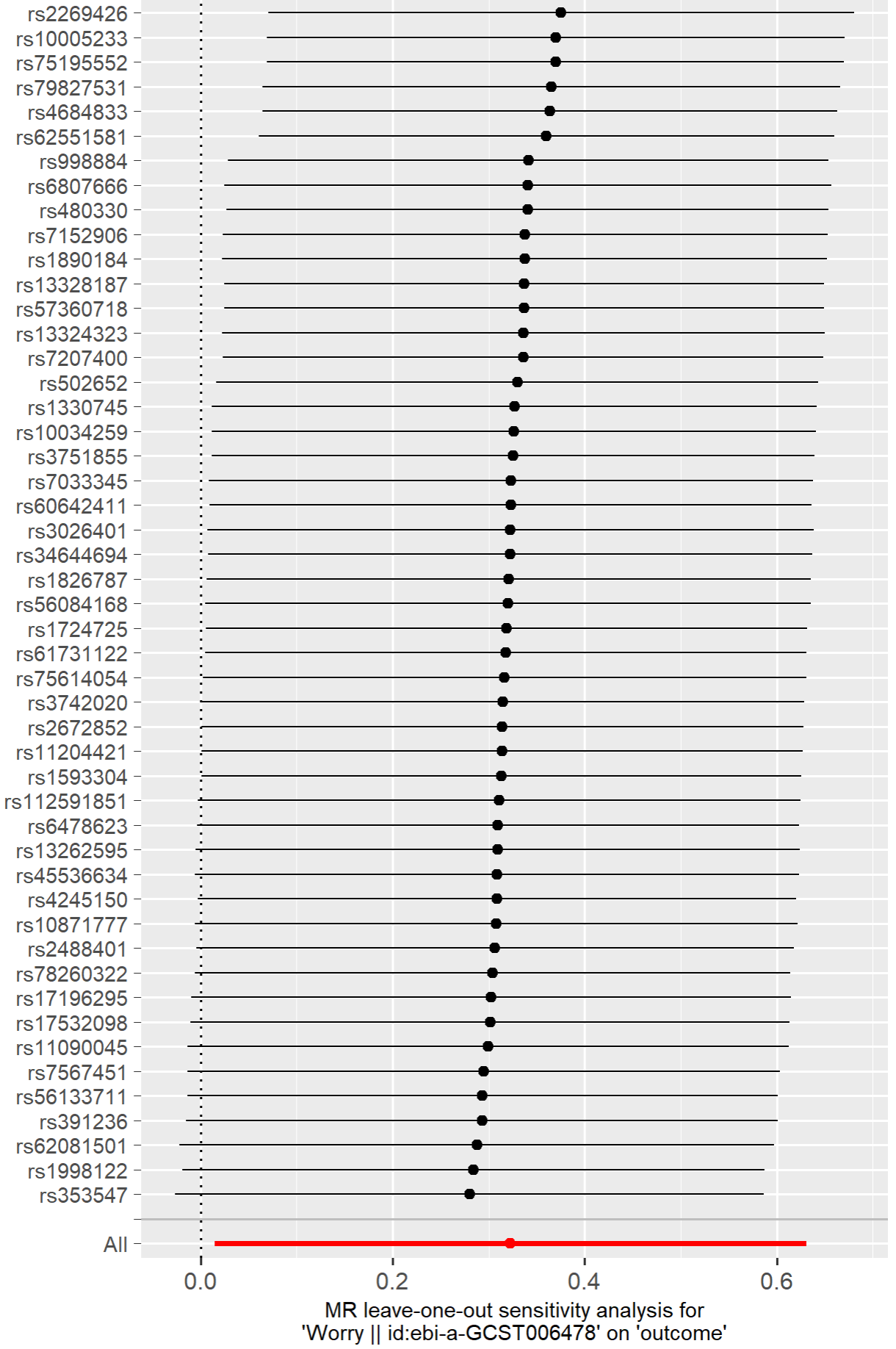


**Supplementary Figure 3.** Scatter plot with lines of different regression analyses of genetic associations (top), and a leave one out analysis (bottom) for inverse variance weighted (IVW) Mendelian randomization estimate, of worry score on probable sleep bruxism.

MR = Mendelian randomization; SNP = single nucleotide polymorphism; rs = reference SNP (ID) cluster

**Depressed Affect**


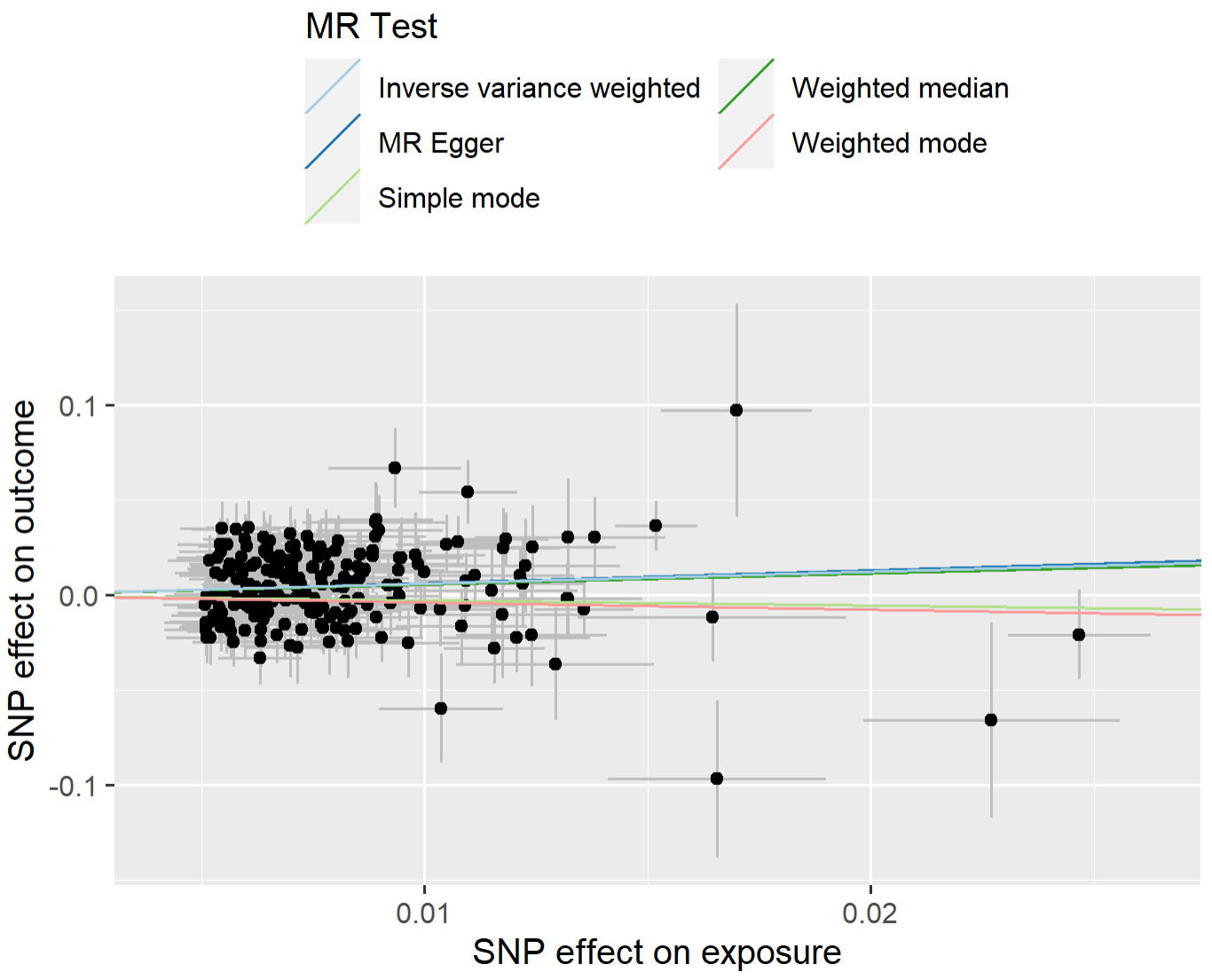


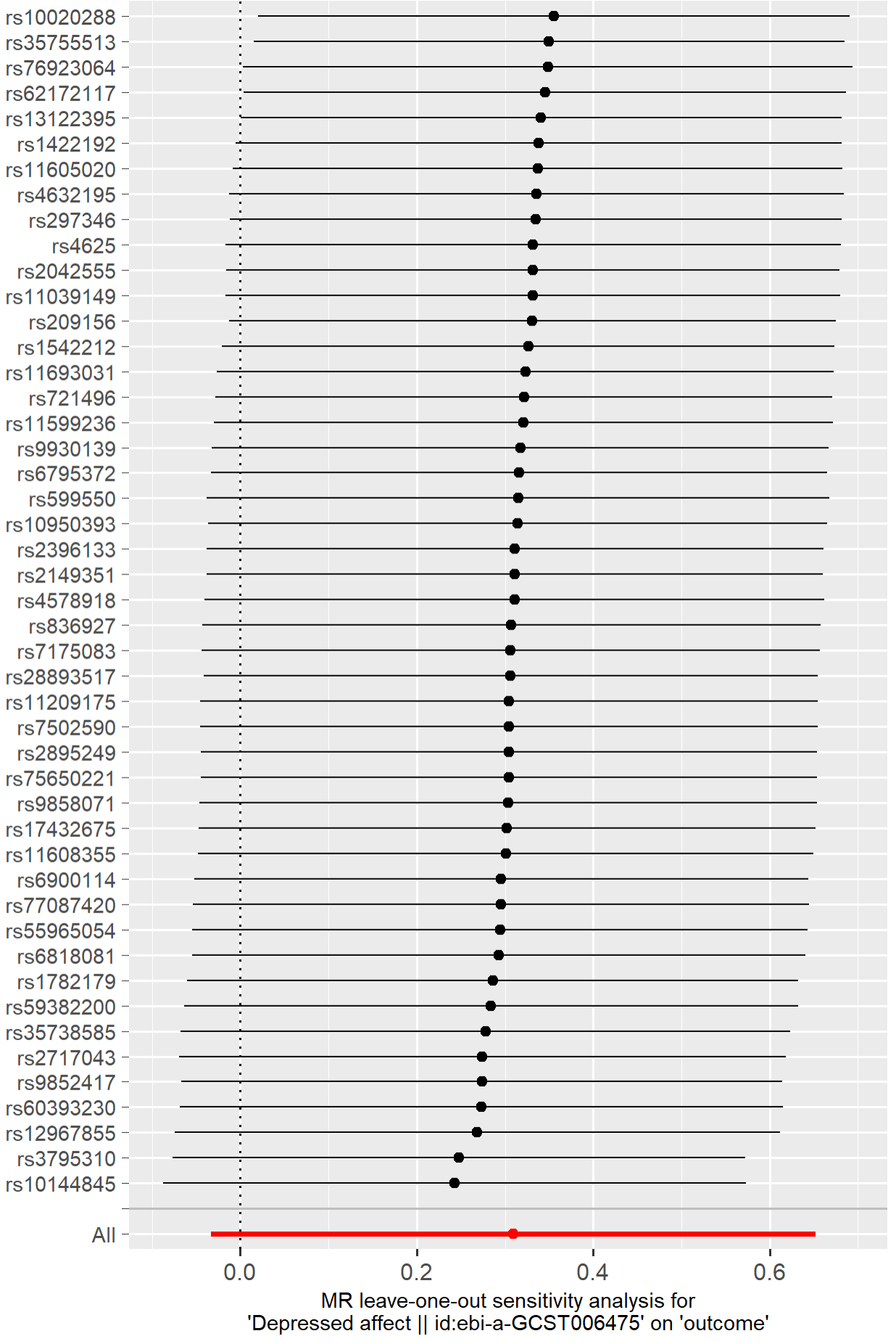


**Supplementary Figure 4.** Scatter plot with lines of different regression analyses of genetic associations (top), and a leave one out analysis (bottom) for inverse variance weighted (IVW) Mendelian randomization estimate, of depressed affect score on probable sleep bruxism.

MR = Mendelian randomization; SNP = single nucleotide polymorphism; rs = reference SNP (ID) cluster

**Sensitivity to environmental stress and adversity (SESA)**


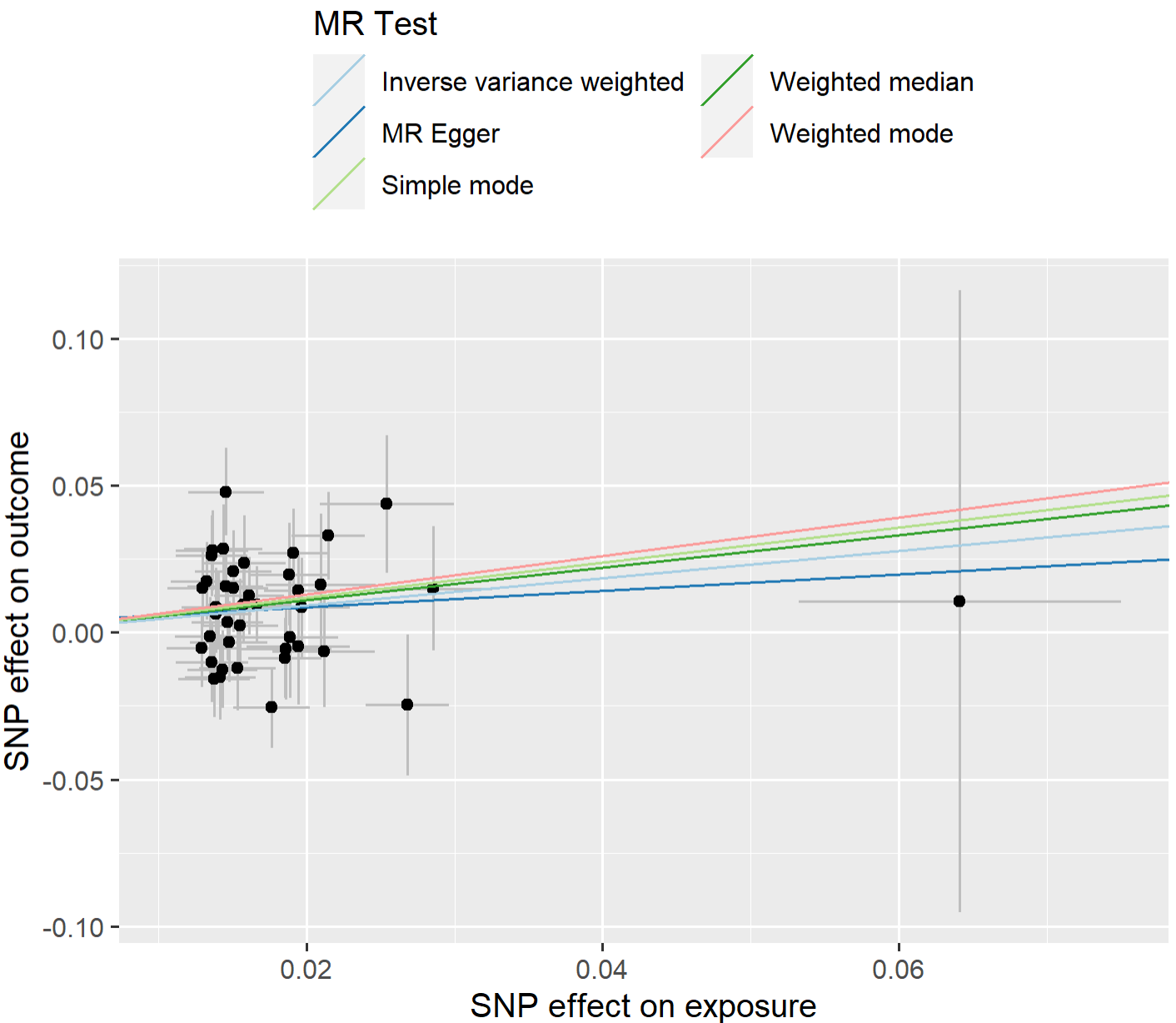


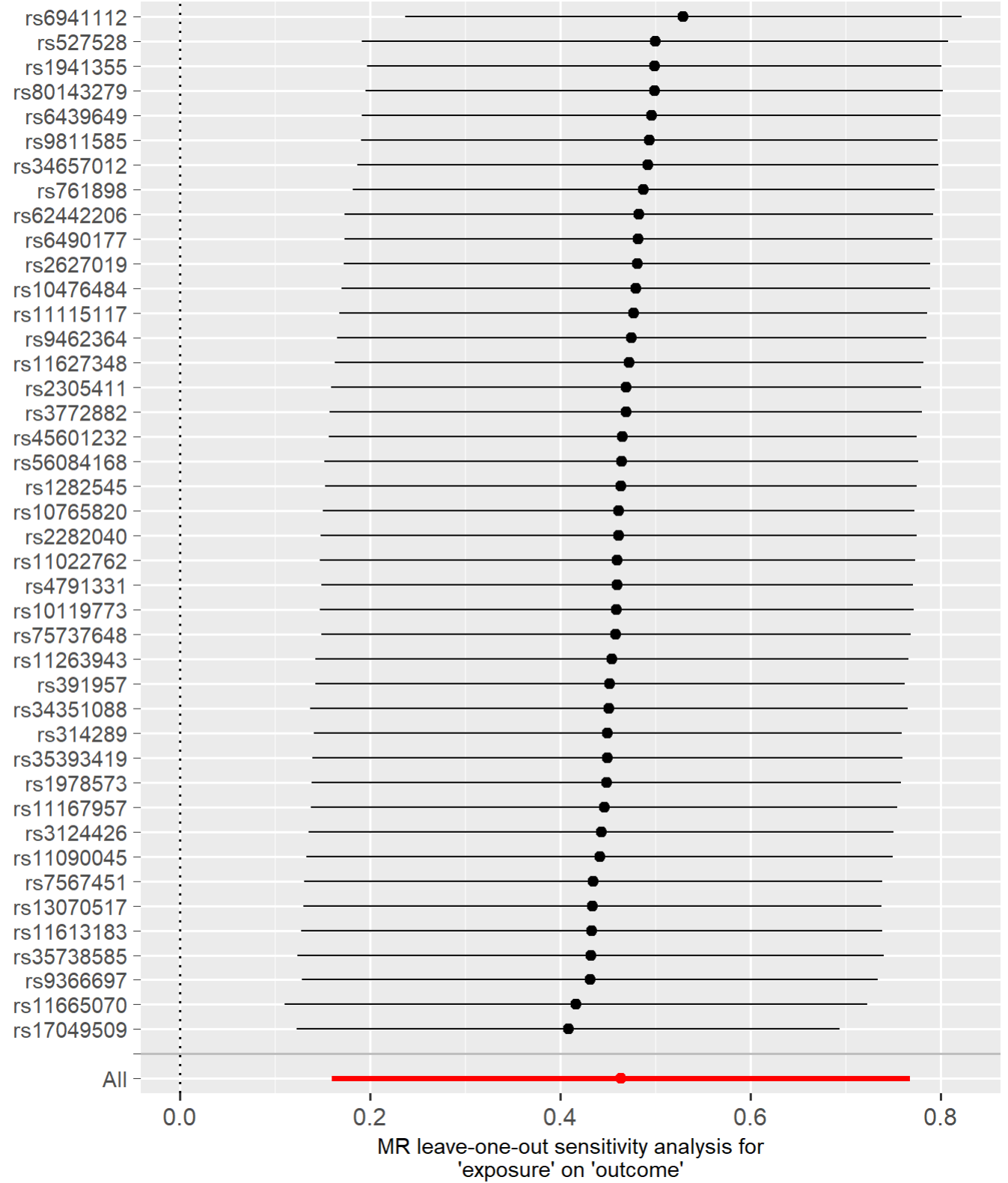


**Supplementary Figure 5.** Scatter plot with lines of different regression analyses of genetic associations (top), and a leave one out analysis (bottom) for inverse variance weighted (IVW) Mendelian randomization estimate, of sensitivity to environmental stress and adversity (SESA) score on probable sleep bruxism.

MR = Mendelian randomization; SNP = single nucleotide polymorphism; rs = reference SNP (ID) cluster
